# Translatome analyses by bio-orthogonal non-canonical amino acid labeling reveal that MR1-activated MAIT cells induce an M1 phenotype and antiviral programming in antigen-presenting monocytes

**DOI:** 10.3389/fimmu.2023.1091837

**Published:** 2023-02-16

**Authors:** Josefine Jakob, Andrea Kröger, Frank Klawonn, Dunja Bruder, Lothar Jänsch

**Affiliations:** ^1^ Cellular Proteomics, Helmholtz Centre for Infection Research, Braunschweig, Germany; ^2^ Institute of Medical Microbiology and Hospital Hygiene, Infection Immunology, Health Campus Immunology, Infectiology and Inflammation, Otto-von-Guericke University Magdeburg, Magdeburg, Germany; ^3^ Immune Regulation, Helmholtz Centre for Infection Research, Braunschweig, Germany; ^4^ Innate Immunity and Infection, Helmholtz Centre for Infection Research (HZI), Braunschweig, Germany; ^5^ Institute of Medical Microbiology and Hospital Hygiene, Molecular Microbiology, Health Campus Immunology, Infectiology and Inflammation, Otto von Guericke University Magdeburg, Magdeburg, Germany

**Keywords:** MAIT cell, MR1-mediated MAIT cell activation, Translatome, BONCAT, antiviral, M1 macrophage polarization, Proteomics

## Abstract

MAIT cells are multifunctional innate-like effector cells recognizing bacterial-derived vitamin B metabolites presented by the non-polymorphic MHC class I related protein 1 (MR1). However, our understanding of MR1-mediated responses of MAIT cells upon their interaction with other immune cells is still incomplete. Here, we performed the first translatome study of primary human MAIT cells interacting with THP-1 monocytes in a bicellular system. We analyzed the interaction between MAIT and THP-1 cells in the presence of the activating 5-OP-RU or the inhibitory Ac-6-FP MR1-ligand. Using bio-orthogonal non-canonical amino acid tagging (BONCAT) we were able to enrich selectively those proteins that were newly translated during MR1-dependent cellular interaction. Subsequently, newly translated proteins were measured cell-type-specifically by ultrasensitive proteomics to decipher the coinciding immune responses in both cell types. This strategy identified over 2,000 MAIT and 3,000 THP-1 active protein translations following MR1 ligand stimulations. Translation in both cell types was found to be increased by 5-OP-RU, which correlated with their conjugation frequency and CD3 polarization at MAIT cell immunological synapses in the presence of 5-OP-RU. In contrast, Ac-6-FP only regulated a few protein translations, including GSK3B, indicating an anergic phenotype. In addition to known effector responses, 5-OP-RU-induced protein translations uncovered type I and type II Interferon-driven protein expression profiles in both MAIT and THP-1 cells. Interestingly, the translatome of THP-1 cells suggested that activated MAIT cells can impact M1/M2 polarization in these cells. Indeed, gene and surface expression of *CXCL10*, *IL-1β*, CD80, and CD206 confirmed an M1-like phenotype of macrophages being induced in the presence of 5-OP-RU-activated MAIT cells. Furthermore, we validated that the Interferon-driven translatome was accompanied by the induction of an antiviral phenotype in THP-1 cells, which were found able to suppress viral replication following conjugation with MR1-activated MAIT cells. In conclusion, BONCAT translatomics extended our knowledge of MAIT cell immune responses at the protein level and discovered that MR1-activated MAIT cells are sufficient to induce M1 polarization and an anti-viral program of macrophages.

## Introduction

Mucosal-associated invariant T (MAIT) cells are multifunctional effector cells, sharing functions from both innate and adaptive immunity. MAIT cells are a common subset of CD3^+^ T cells in humans, accounting for about 1-4% of all CD3^+^ T cells in peripheral blood, up to 10% in the lungs, and even up to 45% of liver lymphocytes (1–3). MAIT cells mediate protection against bacterial infections and sepsis (4) but also contribute to the outcome of viral infections including SARS-CoV-2 (5) and Influenza (6, 7). Importantly, human MAIT cells express a semi-invariant T cell receptor (TCR) Vα7.2 - Jα33/12/20 (1, 8), which allows them to recognize metabolites presented by the Major-Histocompatibility complex class I related protein 1 (MR1) on antigen-presenting cells (APCs). Such MR1 ligands constitute a more recently discovered and growing class of antigens and immune-regulatory molecules (9, 10). Their presentation to the semi-invariant T cell receptor on MAIT cells either activates or inhibits MAIT cell responses, respectively (2, 11, 12). While activating MR1-ligands like 5-(2-oxopropylideneamino)-6-d-ribitylaminouracil (5-OP-RU) are mainly microbial-derived riboflavin-derivatives, inhibitory ligands such as 6-formylpterin (6-FP) or its synthetic analog Acetyl-6-formylpterin (Ac-6-FP) are degradation products from folic acid (13). Interestingly, several drug-like molecules were also identified to interfere with MAIT cell activity (14) and have gained attention as potential therapeutic targets.

Following MR1-dependent activation by exogenous ligands, MAIT cells execute multifunctional effector functions. Among those, MAIT cells can directly kill infected cells by forming an immunological synapse (IS) and releasing lytic granules containing effector molecules such as Granzyme B (GzmB) and Perforin (15). MAIT cells can also secrete pro-inflammatory cytokines such as Interferon-gamma (IFN−γ), Tumor Necrosis Factor-alpha (TNF), and Interleukin-(IL-)17 (2, 6, 16, 17). Due to constitutive expression of the respective cytokine receptors, MAIT cells can as well mediate antiviral responses following cytokine stimulation with e.g. IL-12/-15/-18 and type I Interferon in an MR1-TCR-independent manner (7, 18). Notably, upregulation of a “tissue repair” profile upon TCR stimulation with commensal microbiota indicates that they might have important functions in promoting wound healing and can distinguish between commensal and pathogenic bacteria (16, 19–21).

While tremendous progress has been made in the identification of the multifaceted MAIT cell functions, still a deeper level of understanding of MAIT cell effector processes is crucial for the integration of MAIT cells into immune-regulatory concepts. Different transcriptomic studies have contributed to a more systematic understanding of MAIT cell responses in resting (22, 23) but also activated states (16, 20, 21). Complementary, we used proteomics to describe the unique effector repertoire of MAIT cells of healthy individuals (24, 25). Furthermore, in a multi-OMIC approach, proteomics was used to compare TCR- and cytokine-activated MAIT cell activation (26). However, knowledge of MR1- MAIT responsiveness to MR1-ligands or antigens at the protein level is still missing. Therefore, we here established a translatome approach to study the immune response of human MAIT cells in the presence of specific MR1-ligands presented on APCs. Whereas the proteome defines the steady state of the cells protein inventory, the translatome defines newly synthesized proteins and thus the immune response to a given trigger. Although protein translation is an important regulatory layer, very few translatome studies have been accomplished in T cells (27–30), and none in MAIT cells.

One of the rare technologies suitable to selectivity study translatomes is Bio-orthogonal non-canonical amino acids tagging (BONCAT). BONCAT is based on the cellular uptake of non-canonical amino acids such as L-Azidohomoalanine (AHA) (31, 32). AHA is a methionine analog containing an azide moiety and is incorporated into newly synthesized proteins by the methionyl-tRNA synthetase. Azides are bio-orthogonal, meaning that they do not cross-react with natural biological chemistries (33). However, azides can specifically react with alkynes in a “click” or Cu(I)-catalyzed azide-alkyne cycloaddition reaction, whose scientific impact was recently (2022) awarded the Nobel prize in chemistry (34). The Click reaction allows the specific and covalent binding of AHA-containing proteins to alkyne-bearing beads, enabling a targeted enrichment of translated proteins before measuring them by mass spectrometry (MS). In particular, enrichment reduces sample complexity and facilitates the detection of stimulation-dependent protein translations. Further, the incorporation of AHA has no notable side effects on protein functions and stabilities (31, 35). Although, the identification by BONCAT is limited to methionine-containing proteins, at least 94% of the human proteome is accessible by BONCAT (31, 36, 37).

Here, we now studied MR1-mediated immune responses of MAIT and THP-1 cells in a bicellular system by translatomics. We used BONCAT to selectively enrich only those proteins for mass spectrometric analyses that were translated during the stimulation with MR1-ligands. The subsequent analysis of differentially translated proteins allowed us to define known and novel MR1/TCR-dependent effector responses including the induction of type I Interferon protein profiles in both cell types. Most importantly, the translatome data revealed that 5-OP-RU-activated MAIT cells are able to induce an M1-like phenotype of THP-1 cells, which was found accompanied by the increased antiviral capacity of macrophages.

## Materials & methods

### Synthesis of MR1-ligands

5-OP-RU was kindly provided by Prof. Oliver Lantz (INSERM U932, PSL University, Institut Curie, Paris 75005, France) and synthesized as described (38, 39). Ac-6-FP was commercially purchased by Cayman Chemical.

### Blood donations

This study was conducted in accordance with the rules of the Regional Ethics Committee of Lower Saxony, Germany, and the declaration of Helsinki. Buffy coats from blood donations of healthy human volunteers, who provided informed consent, were obtained from the blood transfusion service Deutsches Rotes Kreuz localized in Springe, Lower Saxony, Germany. Standardized laboratory tests were performed to check blood donors’ health before blood donation. Tests included analysis for infections with HIV1/2, HBV, HCV, and Treponema pallidum (serology and/or nucleic acid testing) and hematological cell counts.

### Cell culture and Fluorescence-Activated Cell Sorting (FACS)

THP-1 acute monocytic leukemia ATCC TIB-202™ (short THP-1) cells were cultured in RPMI complete medium (RPMI 1640 medium (Gibco/Life Technologies) supplemented with 10% fetal bovine serum gold (FCS, PAA Laboratories), 2 mM L-glutamine, 50 units/ml penicillin and 50 µg/ml streptomycin (all Gibco/Life Technologies)) at 37°C in a humid 7.5% CO_2_ atmosphere. Cells were maintained in culture by centrifugation at 125xg for 10 minutes and subsequently resuspended twice per week to a concentration of 200,000 viable cells/ml.

For isolation of Peripheral Blood Mononuclear Cells (PBMCs), buffy coats were produced from whole blood donations by using the Top & Bottom Extraction Bag System (PolymedMedical Devices). PBMCs were isolated from buffy coats by Ficoll^®^ Paque PLUS density gradient centrifugation (GE Healthcare GmbH). PBMCs were rested overnight in RPMI complete medium at 37°C in a humid 7.5% CO_2_ atmosphere. PBMCs with suitable MAIT cell numbers (MAIT cells being >3% of CD3^+^ T cells) were stained for CD3, CD161, and TCR Vα7.2 for 15 minutes at 4°C. MAIT cells were sorted as CD3^+^ Vα7.2^+^ CD161^++^ lymphocytes. Sorted cells were washed with FACS buffer (2% FBS (v/v), 2 mM EDTA in PBS) and rested overnight in RPMI complete medium at 37°C in a humid 7.5% CO_2_ atmosphere.

### Stimulation of MAIT cells

#### Titration of MR1-ligands in the bi-cellular system

All experiments were performed in technical duplicates for each donor. For MR1-ligand titration assays, sorted MAIT cells were plated at 20,000 cells/well in 96-well U-bottom plates and incubated with or without THP-1 cells (50,000 cells/well) in the presence or absence of different concentrations of 5-OP-RU and/or Ac-6-FP (final volume 100 μl, RPMI complete medium). The concentration of used MR1-ligands ranged from 5 ng/ml to 1 µg/ml. Ac-6-FP was added 1 hour before 5-OP-RU and MAIT cells to THP-1 cells. Cells were incubated at 37°C in a humid 7.5% CO_2_ atmosphere. Activation of live MAIT cells was determined after 20 hours by flow cytometry (see chapter Antibodies and extracellular cell staining). For investigation of MR1-dependency of MAIT cell activation, cells were pre-incubated with 20 μg/ml anti-MR1 (Biolegend, clone 26.5) for 1 hour.

#### Conjugation assay

THP-1 cells were loaded with 50 ng/ml 5-OP-RU or Ac-6-FP for 5 hours at 37 °C. The protocol for conjugation was adapted from (40). After MR1-loading, THP-1 cells were stained with 5 µM CellTracker™ Green CMFDA Dye (Life Technologies) and FACS sorted MAIT cells with 5 µM CellTracker™ Blue CMAC Dye (Life Technologies) according to the manufacturer instructions. MAIT and THP-1 cells were paired in a one-to-one ratio at 37 °C and conjugate formation was assessed at indicated time points by flow cytometry as described in 40.

#### Immunofluorescence microscopy

THP-1 cells were loaded with 50 ng/ml 5-OP-RU or Ac-6-FP for 5 hours at 37 °C. THP-1 cells were washed with RPMI complete medium containing 1% FCS (RPMI1640 with 1% FCS) and paired with FACS-sorted MAIT cells in RPMI 1% FCS (ratio 3:2). Cell suspensions containing MAIT and THP-1 cells were immediately seeded onto poly-L-lysine (Sigma) coated coverslips and allowed to conjugate and adhere for 30 minutes. Cells were fixed by using 4% paraformaldehyde (Sigma) in PBS for 20 minutes followed by washing with PBS. Cells were permeabilized using 0.15% Triton-X100 (Sigma) in PBS for 5 minutes followed by blocking using 1% BSA (Sigma) in PBS containing 0.05% Tween-20 (Roth) for 1 hour. Anti-CD3ϵ antibody (eBioscience, clone OKT3) was applied to the blocking solution, and staining was performed for 2 hours at room temperature (RT). Cells were washed three times with PBS containing 0.05% Tween-20. Staining with goat anti-Mouse IgG secondary antibody (Alexa488, Invitrogen, A11029) and Phalloidin (ATTO594, ATTO-TEC) using 1% BSA in PBS was performed for 1 hour at RT. Cells were washed three times with PBS containing 0.05% Tween-20 and three times in pure PBS, followed by dehydration using first 70% and then 100% ethanol. Samples were dried by air and mounted using Mowiol (Roth). Light microscopy was carried out on an inverted microscope (ECLIPSE Ti-E; Nikon) with standard epifluorescence illumination (Intensilight C-HGFIE; Nikon) and 100×/NA1.4 plan-apochromatic objective. Images were acquired as z-stacks from the immunological synapse with a back-illuminated, cooled charge-coupled device camera (DS2-Qi2; Nikon) driven by NIS-Elements (Nikon). Data acquisition was performed in NIS-Elements. Z-stack images were combined with extended depth of field (EDF) focused images.

#### Quantitative gene expression analyses by RT-qPCR

450,000 MAIT cells and 1,125,000 THP-1 cells were stimulated with 50 ng/ml 5-OP-RU or Ac-6-FP (in 1500 µl, 12 well plate format) for 20 hours at 37°C. An aliquot was taken for verification of MAIT cell activation by flow cytometry as described below. MAIT cells were sorted as CD3^+^ Vα7.2^+^ CD161^++^ cells and THP-1 cells were separated by forward scatter-area (FSC-A) against side scatter-area (SSC-A) from MAIT cells. RNA was isolated using RNeasy^®^ Mini Kit (Qiagen). Subsequently, 1 µg of total RNA was reverse-transcribed with anchored oligo(dT)18 primers, random hexamer primers, and 10 U Transcriptor reverse transcriptase using Transcriptor First Strand cDNA Synthesis Kit (Roche). cDNA was analyzed by qPCR (Meridian Bioscience™) using primers shown in **Table 1**. Relative mRNA expression was calculated using LightCycler^®^480 Software (Roche).

**Table 1:** Primer sequences used for RT-qPCR (synthesized by Eurofins Genomics).

Gene	Forward Primer sequence	Reverse Primer sequence
GAPDH	GGATTTGGTCGTATTGGGCG	ATGGAATTTGCCATGGGTGG
IL1β	GAAATGATGGCTTATTACAGTGGC	TAGTGGTGGTCGGAGATTCG
CXCL10	GTCCACGTGTTGAGATCATTGCTA	AGCACTGCATCGATTTTGCTC
Rps9	GAAATCTCGTCTCGACCAAGAG	GGTCCTTCTCATCAAGCGTCA
IL12A	ATGGCCCTGTGCCTTAGTAGT	AGCTTTGCATTCATGGTCTTGA
IL15	TTTCAGTGCAGGGCTTCCTAA	GGGTGAACATCACTTTCCGTAT
IL18	TCTTCATTGACCAAGGAAATCGG	TCCGGGGTGCATTATCTCTAC

#### Polarization of primary macrophages

MAIT cells and CD14^+^ monocytes were sorted by FACS as described above. MAIT cells were frozen in liquid nitrogen in FCS supplemented with 20% DMSO until the end of the macrophage differentiation. 200,000 monocytes were plated in a 24-well plate in 750 µl RPMI complete medium supplemented with 50 ng/ml M-CSF (Miltenyi Biotec) and differentiated for 8 days into M0 phenotype. Medium containing M-CSF was replaced on days 3 and 5. On day 7, a medium without M-CSF was added and MAIT cells were thawed and cultured overnight in RPMI complete medium. On day 8, MAIT cells were added to adherent macrophages and stimulated with 50 ng/ml 5-OP-RU or Ac-6-FP for 20 hours at 37 °C. As positive controls, M0 macrophages were stimulated for 20 hours with 20 ng/ml IFN-γ (PeproTech), IL-4 (PeproTech), or LPS (Sigma-Aldrich). After stimulation, cells were stained for live/dead cells as well as CD14, CD80, CD206, and CD69 using extracellular cell staining as described below and measured by flow cytometry. IFN-γ was determined in the supernatants using Human IFN-γ LEGEND MAX™ ELISA Kit (Biolegend).

#### Production of VSV-eGFP

Vesicular-stomatitis virus expressing GFP (VSV-eGFP) was produced in BHK-21 cells and harvested from cell culture supernatants as described before in 41. A viral titer of 1.5x10^6^ Plaque forming units/well was determined by plaque formation on Vero cells. Serial 10-fold dilutions of virus stocks were transferred onto Vero cell monolayers in six-well plates and incubated for 1 hour at 37 °C. Monolayers were overlaid with 2 ml of MEM containing 1% methylcellulose. Upon plaque formation cells were fixed and stained using crystal violet solutions.

#### Infection with VSV-eGFP

MAIT cells were sorted by FACS as described above. THP-1 cells and sorted MAIT cells were paired in a one-to-one ratio and stimulated with 5 or 50 ng/ml 5-OP-RU and 50 ng/ml Ac-6-FP at 37°C for 20 hours. For assessment of IFN-γ- and MR1-dependency, THP-1 cells were pre-incubated with 20 µg/ml anti-MR1 or MAIT and THP-1 cells with 5 µg/ml anti-CD119 1 hour before stimulation with 5-OP-RU. After stimulation, cells were washed with a 0% FCS RPMI medium, and the cell number of each condition was determined using a cell counting chamber. MAIT cell activation in each condition was quantified by flow cytometry, staining an aliquot for live/dead cells, and CD69 as described for extracellular cell staining below. Unstained cells were stimulated at MOI of 5 with VSV-eGFP virus in 0% FCS RPMI medium and incubated at 37 °C. After 60 minutes the cells were centrifuged and the supernatant was removed. Cells were resuspended in RPMI complete medium and cultured for 5 hours at 37 °C. Finally, cells were washed with PBS and stained with Fixable viability dye LIVE/DEAD™ Cell Stain Kit (Invitrogen™, 34975), before measuring GFP fluorescence by flow cytometry. As a positive control, THP-1 cells were pre-incubated with 500 U/ml Interferon-(IFN-)αB/D (42) for 24 hours before infecting them with VSV-eGFP. IFN-αB/D was kindly provided by Peter Stäheli (Virology, University of Freiburg, Freiburg, Germany). For MR1-monomer-dependent activation, a flat-bottom 96-well plate was coated with 5 µg/ml Strepatividin (Biolegend) in 0.05 M Carbonate binding buffer overnight at 4°C. Streptavidin-coated wells were first washed with PBS+0.05% Tween-20 (Sigma-Aldrich) and then PBS only. 500 ng MR1-5-OP-RU- or MR1-Ac-6-FP-monomer per well were coated overnight at 4°C in PBS. MR1-monomer-coated wells were washed two times with PBS. FACS-sorted MAIT cells were added for 2 hours together with 1 µg/ml anti-CD28 (Biolegend, clone CD28.2). After MR1-monomer stimulation, MAIT cells were co-cultured with naïve THP-1 cells for 20 hours at 37°C and infected with VSV-eGFP as described above.

#### Antibodies and extracellular cell staining

For flow cytometric assessment of MAIT cell or macrophage phenotypes, cells were stained with Fixable viability dye LIVE/DEAD™ Cell Stain Kit (Invitrogen™), with Fc receptor blocking reagent (Miltenyi Biotec) and combinations of the following antibodies (from BioLegend except as noted): CD3 BV605 (clone UCHT1, Becton Dickinson), CD161 APC (clone DX12, Becton Dickinson), Vα7.2 PE-Cy7 (clone 3C10), CD69 PE or APC (clone FN50), CD14 BV421 (clone 3D3), CD206 FITC (clone MMR), CD80 PE (2D10). For staining of differentiated macrophages, cells were detached after washing with PBS using 0.05% Trypsin –EDTA (ThermoFisher Scientific, 25300-054) for 3 minutes at 37°C. All other cells were directly washed with PBS after stimulation and stained with Fc receptor blocking reagent (Miltenyi Biotec) and LIVE/DEAD™ Fixable Blue/Near-IR Dead Cell Stain Kit (Invitrogen) for 10 minutes at 4°C. Cells were washed with PBS and stained for extracellular surface markers at 4°C for 30 minutes in FACS Buffer. Cell fixation was performed by resuspending cells in 2% paraformaldehyde in PBS for 10 minutes at 4°C. Cells were washed with PBS and the cell pellet was resuspended in FACS buffer and subsequently analyzed on MACSQuant^®^ Analyser 10 or BD LSR-Fortessa flow cytometer. Data were analyzed by FlowJo (TreeStar, v10.8.0) and Prism (GraphPad Software, v9.3.0). To determine significant differences, a one-tailed Wilcoxon matched-pairs signed rank test was used.

#### Translatome analysis of MAIT and THP-1 cells

For translatome analysis of the bicellular system, we isolated MAIT cells from 6 healthy human donors by FACS as described above. The labeling of proteins during their translation was realized by co-culturing 400,000 sorted MAIT cells and 1,000,000 THP-1 cells in 1 mM AHA medium (RPMI1640 without methionine supplemented with 1 mM AHA, 10% dialyzed FCS, 2 mM L-glutamine, 50 units/ml penicillin and 50 µg/ml streptomycin). Cells were treated with 50 ng/ml 5-OP-RU, 50 ng/ml Ac-6-FP, or a MOCK-control for 20 hours (7.5% CO_2_, 37°C). For the monocellular system, equal cell numbers and stimulation conditions were used but MAIT cells of 4 different donors and 3 THP-1 replicates were cultured in absence of the respective other cell type.

After stimulation, an aliquot was taken for verification of MAIT/THP-1 cell viability and MAIT cell activation by flow cytometry as described above. MAIT and THP-1 cells from the bicellular system were separated by FACS as described for gene expression analyses by RT-qPCR above. Cells were lysed and AHA-containing proteins were enriched using the Click-iT^®^ Protein Enrichment Kit (Invitrogen™). Translated proteins were separated from resin beads using 10 μM MobiSpinColumn filters (MoBiTec) after tryptic digest. Peptides were further processed for proteomic analysis using single-pot, solid-phase-enhanced sample preparation (SP3, 43). The binding of peptides to SP3 carboxylated beads was enabled using 95% Acetonitrile (ACN) while incubating the samples overnight at room temperature at 700 rpm. SP3 beads were washed with ACN and the supernatant was incubated again with SP3 beads overnight to ensure complete binding of all peptides. Beads were washed two times with ACN and air-dried at room temperature. Peptides were eluted from beads first with 2% DMSO and second with ddH_2_O. Samples were dried and solved in 0.2% trifluoroacetic acid/3% ACN. Samples were ultracentrifuged for 20 minutes at 50,000xg before measuring them by LC-MS/MS on timsTOF™Pro (Bruker Daltonik GmbH, Application Version 6.2.0.7) coupled to the High-Pressure Liquid Chromatography (HPLC) system EvosepOne (Evosep). The default method by EvosepOne for short gradients with 60 samples per day was used for HPLC separation (44). All spectra were acquired using Compass Data Analysis v5.3 (Bruker). The MS/MS raw data files were processed using PEAKS Studio 10.6 using Label-free Quantification (LFQ). Peptides were identified using the UniprotKB/Swiss-Prot protein human database and contaminant database (Accessed Dec 2020, http://lotus1.gwdg.de/mpg/mmbc/maxquant_input.nsf/7994124a4298328fc125748d0048fee2/$FILE/contaminants.fasta). Carbamidomethylation was set as fixed, and oxidation of methionine as variable modification. If not stated otherwise, default parameters of PEAKS Studio 10.6 were used. Parent and fragment mass error tolerance was set as 20 ppm/0.3 Da. Only peptides with a retention time between three and 21 min and a charge of [2-4] were selected for data analyses. Furthermore, only peptides with none or maximal one missed cleavage site and three variable posttranslational modifications were considered for protein identification and quantification. The main steps of the translatome workflow are also summarized in **Supplementary Figure S3**.

The specificity of the Click-reaction (Enrichment controls) was validated by culturing MAIT cells and THP-1 cells in normal RPMI complete medium (no AHA) and used to determine low-confident/non-AHA-specific protein translations (see next chapter). The stimulation, sorting, and Click-Reaction were performed as described above. In addition, click-reaction efficiency was determined by analyzing AHA residues in the translatome data. Click-reaction efficiency was at least 99% in this study.

AHA incorporation was validated by measuring AHA within the whole proteome lysate by FUNCAT (fluorescent noncanonical amino acid tagging). For this purpose, cells of two MAIT donors were stimulated with THP-1 cells as described above for 20 hours in AHA medium. After stimulation, MAIT and THP-1 cells were separated by FACS and lysed as described above. Subsequently, AHA-containing proteins were covalently coupled to an Alkyne-linked fluorophor (AF488, Jena Bioscience-Alkyne) using the CuAAC Biomolecule Reaction Buffer Kit (BTTAA based, Jena Bioscience). Fluorescent lysates were then separated on a 10% SDS gel for 90 minutes, proteins in the gel were fixed for 30 minutes using 40% ddH_2_O/10% acetic acid/50% EtOH, and AF488 fluorescence was measured with Typhoon™ FLA 9000 (GE Healthcare). Finally, a Coomassie stain of the gel was performed overnight to normalize AF488 fluorescence to the protein amount.

#### Evaluation of proteomic data and statistical analysis

Protein intensities, which were calculated by LFQ based on the top three peptides per protein, were processed with Perseus 1.6.15.0 and Rv4.0.2 (45) in R studio (45) with packages tidyverse (46), openxlsx (47), ggrepel (48), rstatix (49), ggpubr (50), ggplot2 (51), tibble (52) (Accessed on Jan 2021). In brief, protein contaminants were excluded and data were grouped into the 3 stimulation conditions: unstimulated, 5-OP-RU, and Ac-6-FP. Protein translations were considered robust in primary MAIT and THP-1 cells, if protein groups were detected in at least 4 out of 6 donors (or 3 out of 4 donors in the monocellular system). Protein groups that were exclusively found in one condition were determined using Venn diagrams that were calculated with Perseus. To analyze the regulation of translated proteins, intensities of protein groups were log_2_-transformed and missing values were replaced from a normal distribution with default parameters of Perseus. Subsequently, fold changes (FCs) were calculated in comparison to unstimulated samples using the median of intensities per group. P-values were calculated using the Student’s paired t-test in Rv4.0.2. Proteins with a p-value ≤ 0.05 and lg_2_(FC)>[1] were considered differentially regulated. Regulated protein translations were analyzed at the level of pathways using the Reactome database (53). Enrichment controls (no AHA) were processed as described before to determine proteins that were not AHA-selectively clicked. Proteins identified in those controls, which were equally regulated in the absence, and presence of AHA were considered low-confident protein translations. Low-confident protein translations were marked in red in **Supplementary Tables 1**, **2** and not included in pathway analysis and data interpretation. Pathway analysis was performed by analyzing differentially upregulated proteins (except low-confident proteins) using the Reactome database (53). Lowest p-values indicate the most affected pathways. Graphs and statistical analyses of proteomic results were completed using Rv4.0.2 in R studio.

## Results

### 5-OP-RU, but not Ac-6-FP, increases conjugation of primary MAIT cells with THP-1 cells

**Figure 1:**
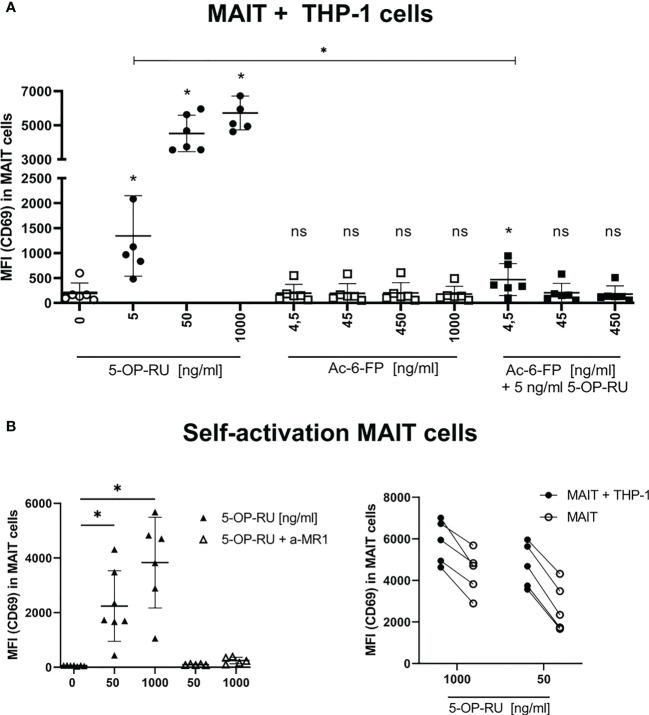
Metabolite-induced MAIT cell activation. **(A)** FACS-sorted primary human CD161^++^ Vα7.2^+^ CD3^+^ MAIT cells were paired with THP-1 cells (2:5 ratio) and stimulated with increasing concentrations of 5-OP-RU and Ac-6-FP for 20 hours at 37°C. For simultaneous stimulation with both metabolites, cells were pre-incubated for 1 hour at 37°C with varying concentrations of Ac-6-FP before 5 ng/ml 5-OP-RU were added. (**B**, left) MAIT cells were stimulated without THP-1 cells with indicated 5-OP-RU concentrations. Anti-MR1 (a-MR1) was added 1 hour prior to stimulation and incubated at 37°C. (**B**, right) Comparison of MAIT cell activation from bi- and mono-cellular system after stimulation with 5-OP-RU. Lines connect results from the same donors. (**A, B**) MAIT cell activation was quantified by flow cytometry determining the median fluorescence intensity (MFI) of CD69 in live MAIT cells. Data from three independent experiments from six donors are shown. Asterisks indicate significant differences determined by Wilcoxon matched-pairs signed rank test: p* < 0.05. Horizontal lines indicate mean ± SD. ns, not significant.

The primary aim of this study was the characterization of MR1-ligand-specific MAIT cell immune responses at the translatome level and at the same time the analysis of the respective translatome response induced in the ligand-presenting cells that are in intimate contact with the MAIT cells. For this purpose, we established a bicellular system consisting of primary human MAIT cells and THP-1 cells as APCs in which we analyzed protein translation by BONCAT proteomics. More specifically, CD3^+^/Vα7.2^+^/CD161^++^ MAIT cells were FACS-sorted from PBMCs of healthy human donors (**Supplementary Figure S1**) and subsequently co-cultured with THP-1 cells in the presence of the MR1-binding ligands 5-OP-RU and Ac-6-FP, respectively. MAIT cell activation was assessed by analyzing the surface expression of CD69 on MAIT cells after 20 hours of stimulation by flow cytometry. As expected, MAIT cells showed a concentration-dependent activation by 5-OP-RU, whereas Ac-6-FP did not induce CD69 expression on MAIT cells (**Figure 1A**). As shown before (13), Ac-6-FP efficiently interfered with the 5-OP-RU-induced MAIT cell activation. Of note, while 10 nM 5-OP-RU typically induce strong activation of MAIT cells within PBMCs (**Supplementary Figure 2A** 20, 54), MAIT cell activation by THP-1 cells in the bicellular system required a higher concentration of 5-OP-RU. We compared MAIT cell activation in human PBMCs with 10 nM 5-OP-RU to the activation in the bicellular system. We observed a comparable MAIT cell activation at 50 ng/ml (=150 nM) 5-OP-RU and thus decided to continue with this concentration for further experiments. We hypothesize that lower 5-OP-RU concentrations in PBMC fractions are usually sufficient because multiple cell types are present and synergistically interact to enhance MR1-MAIT-related responses. The viability of both MAIT and THP-1 cells in the bicellular system was routinely checked for every experiment by flow cytometry. The viability of both cell types was high (>90%) and did not vary between different stimulation conditions. In addition, we analyzed MAIT and THP-1 cell apoptosis in the bicellular system 20 hours following stimulation with 50 ng/ml 5-OP-RU or Ac-6-FP. We did not observe any difference in live (7AAD^-^, AnnexinV^-^), early (7AAD^-^, AnnexinV^+^), and late apoptotic cells (7AAD^+^, AnnexinV^+^) (data not shown). To further characterize MAIT cell activation in the bicellular system, we quantified the concentration of *Interleukin (IL)12*, *IL15*, and *IL18* by RT-qPCR in THP-1 cells (**Supplementary Figure S2B**). We did not observe an increase in *IL18* mRNA expression whereas *IL12A* was moderately and *IL15* mRNA expression was more significantly elevated upon stimulation with 5-OP-RU. However, mRNA expression remained stable for all three interleukins when THP-1 cells were stimulated with MR1-ligands in absence of MAIT cells.

To investigate the impact of co-cultivation with THP-1 cells on MAIT cell activation, we further examined the self-reactivity of MAIT cells in the presence of 5-OP-RU. We indeed observed concentration- and MR1-dependent activation of MAIT cells following 5-OP-RU treatment even in absence of THP-1 cells (**Figure 1B**). Nevertheless, MAIT cell activation as indicated by the expression of CD69 was generally lower in absence of THP-1 cells.

**Figure 2:**
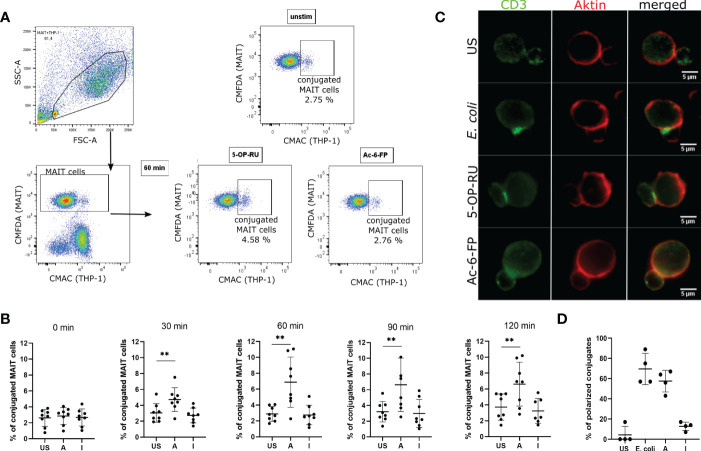
Conjugation frequency between MAIT and THP-1 cells in presence of different MR1 ligands. **(A)** THP-1 cells were loaded with 50 ng/ml 5-OP-RU or Ac-6-FP for 5 hours at 37 °C. FACS-sorted MAIT cells were stained with CMFDA cell tracker dye whereas metabolite-stimulated THP-1 cells were stained with CMAC cell tracker dye. Subsequently, MAIT and THP-1 cells were paired (1:1 ratio) and conjugate formation was analyzed after indicated time points by flow cytometry. MAIT-THP-1-conjugates were identified as CMFDA^+^CMAC^+^ cells. The frequency of conjugated MAIT cells was determined. **(B)** Frequency of conjugated MAIT cells with THP-1 cells is shown after different periods of pairing. Data from four independent experiments from eight donors are shown. Asterisks indicate significant differences determined by Wilcoxon matched-pairs signed rank test; p** < 0.01. Horizontal lines indicate mean ± SD. **(C)** MR1-loaded THP-1 cells were paired with FACS-sorted MAIT cells for 30 minutes on poly-L-lysine coated coverslips at 37 °C. Cells were stained for CD3 (green) and actin (red). Representative images of conjugates are shown after focusing stacked images by extended depth of field (edf). **(D)** Quantification of CD3 polarization towards MTOC in MAIT cells conjugated to THP-1 cells (n=4 donors; at least 32 conjugates were analyzed per condition). US = unstimulated; A = 5-OP-RU; I = Ac-6-FP.

Next, we quantified the conjugation frequency of MAIT and THP-1 cells in the presence or absence of MR1 ligands. To this end, we pre-incubated THP-1 cells with the MR1 ligands 5-OP-RU (activating=A) or Ac-6-FP (inhibiting=I), or left them unstimulated (=US) as internal control, stained MR1-loaded THP-1 cells and primary MAIT cells with individual cell tracker dyes and paired them for 0 to 120 minutes (adapted from 40). MAIT-THP-1-conjugates were then identified as events being positive for both cell tracker dyes by flow cytometry (**Figure 2A**). Although both ligands were shown before to upregulate MR1 expression on the surface of THP-1 cells (13), only 5-OP-RU increased the conjugate formation between MAIT and THP-1 cells, reaching a peak frequency after 60 minutes and remained stable even after 120 minutes of pairing (**Figure 2B**). Since antigen-dependent conjugation goes along with immunological synapse (IS) formation, we analyzed the CD3 polarization of MAIT cells by immunofluorescence microscopy. As a positive control, we used *E.coli*-loaded THP-1 cells that induced polarization of CD3 towards the immunological synapse (IS). While a similar efficient polarization of CD3 was observed with 5-OP-RU, conjugate formation between Ac-6-FP-loaded THP-1 and MAIT cells did not exceed that observed with unloaded THP-1 cells (**Figures 2C, D**). Thus, CD3 polarization at the IS was consistent with results for the conjugate abundance, and responses in the bicellular system are primarily dependent on contact with 5-OP-RU. However, it remains unclear whether or not Ac-6-FP is able to stimulate cellular responses even in the absence of an IS.

### 5-OP-RU induces pro-inflammatory and type I Interferon-driven protein translation both in MAIT and THP-1 cells

Having defined the intercellular crosstalk in the presence of 5-OP-RU and Ac-6-FP, we next analyzed intracellular translatome responses in both cell types. For this purpose, we isolated primary human MAIT cells from six healthy donors. Cells from each donor were co-cultured with THP-1 cells and MR1-ligands in BONCAT (AHA-containing) medium allowing, after sorting, the subsequent enrichment and analyses of protein translations by LC-MS/MS (**Supplementary Figure S3**). AHA incorporation efficiency was validated in whole proteome lysates using FUNCAT (**Supplementary Figure S4**). We used BONCAT to reduce sample complexity in favor of increased sensitivity of mass spectrometry to characterize AHA-containing and stimulation-dependent protein translations. After stimulation, all samples that were processed for translatome analysis were examined for MAIT cell activation and MAIT and THP-1 cell viability by flow cytometry (**Supplementary Figure S5**). Differentially translated proteins were analyzed to examine MR1-ligand-induced responses of both cell types. In addition, proteins that were exclusively detected in one condition were determined since they potentially represent a pool of newly translated candidates.

Under bicellular conditions, but in the absence of MR1-ligands, 933-1230 MAIT cell protein translations (protein groups) per donor could be identified (**Supplementary Figures S6A**, **B**). Following 5-OP-RU-induced stimulation of MAIT cells within the bicellular system, the numbers of protein translations in MAIT cells increased notably (1466-2130 protein groups), demonstrating a global up-regulation of protein synthesis in MR1-activated MAIT cells. In contrast, protein translations in MAIT cells following Ac-6-FP stimulation were comparable to unstimulated cells. In THP-1 cells co-cultured with MAIT cells in absence of MR1-ligands, we identified 2830-3485 protein translations. Interestingly, the number of protein translations remained largely stable following stimulation with 5-OP-RU and Ac-6-FP, respectively. Among all donors and stimulation conditions, we observed 2263 protein translations for MAIT cells and 3254 protein translations for THP-1 cells in the bicellular system (**Table 2**). Next, translatome data were analyzed by Sammon mapping to characterize clustering between our samples (**Supplementary Figures S6C**, **D**). In agreement with the number of protein translations (S6A+B), 5-OP-RU-activated MAIT cells can be distinguished from unstimulated/Ac-6-FP-stimulated samples while no difference was observed for THP-1 cells.

**Table 2:** Regulated protein translations of interacting MAIT and THP-1 cells in the presence of MR1-ligands.

	Stimulation	Upregulated	Downregulated	Proteins total
MAIT cells	5-OP-RU	690	6	2263
	Ac-6-FP	12	11	
THP-1 cells	5-OP-RU	47	18	3254
	Ac-6-FP	3	5	

The total number of regulated protein translations is given for proteins, which were identified in four out of six donors. Differentially abundant proteins were defined with p<0.05 and log_2_ (FC)>[1] in comparison to unstimulated samples.

**Figure 3:**
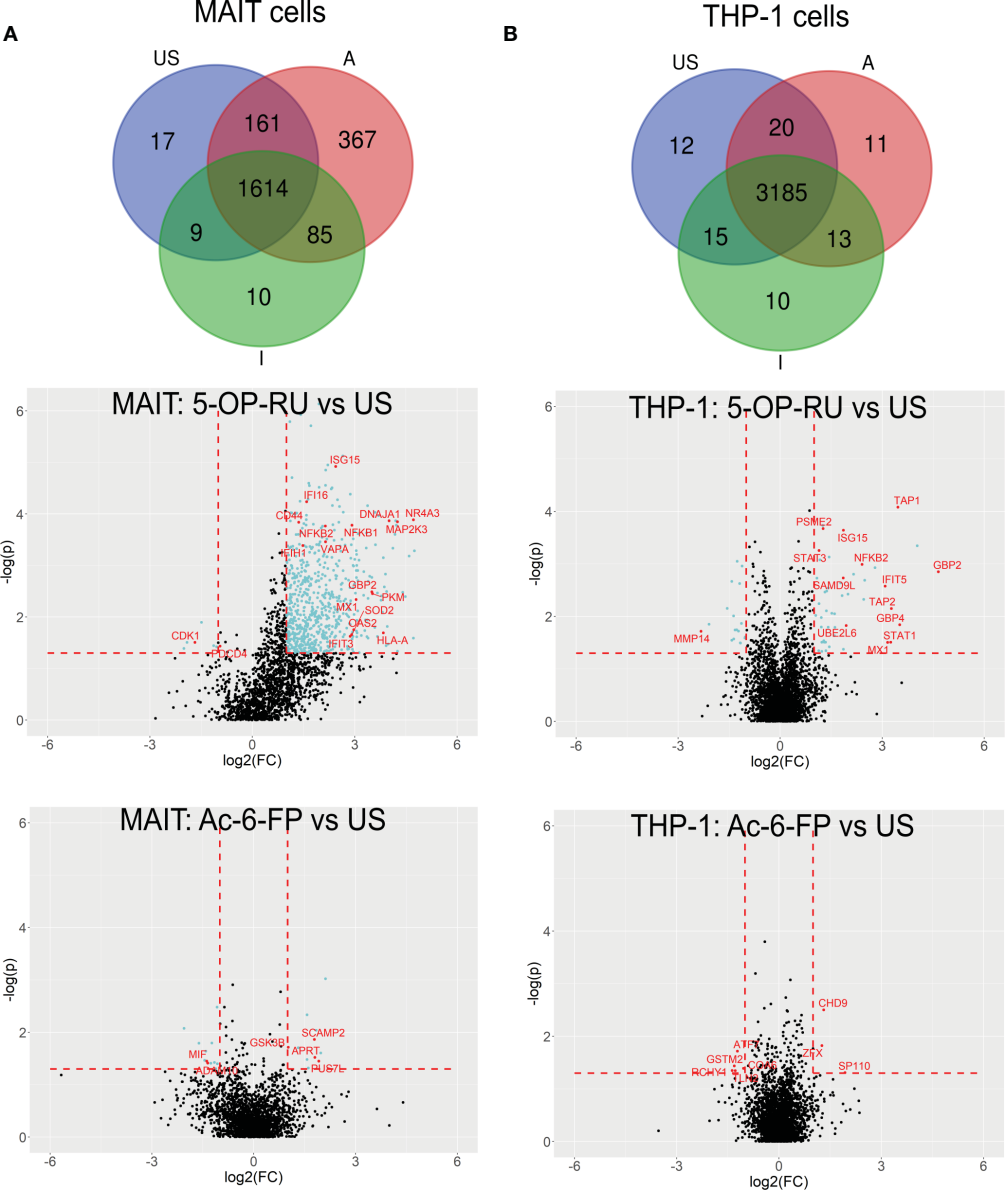
5-OP-RU-activated MAIT and THP-1 cells reveal a distinct pro-inflammatory translatome profile. Top: Venn diagrams showing MAIT **(A)** and THP-1 **(B)** proteins that were identified in each condition in at least four of six donors from the bicellular system. Bottom: Volcano plots visualizing differentially abundant proteins of primary human MAIT cells **(A)** and THP-1 cells **(B)** from the bicellular system. Differentially abundant proteins (p<0.05; log_2_(FC)>[1]) are highlighted in light blue. Most abundant proteins or proteins supporting the pro-inflammatory translatome profile are labeled with their gene names in red. Data from three independent experiments from six donors are shown. US=unstimulated; A=5-OP-RU; I= Ac-6-FP.

For more detailed data evaluation, we first determined and classified proteins that were exclusively identified in one of the stimulation conditions and therefore represent proteins potentially newly translated in response to stimulation with one of the MR1-ligands. Interestingly, 367 proteins were exclusively detected after MAIT cell activation with 5-OP-RU, whereas only 10 proteins were exclusively found to be newly translated after Ac-6-FP stimulation, and 17 in unstimulated samples (**Figure 3A**; **Supplementary Table 5**). To further categorize the 367 5-OP-RU-induced proteins at the level of pathways, we performed an enrichment analysis using the Reactome database (**Supplementary Figure S7A**) (53). This analysis revealed that the four most relevant pathways (highest p-values) were related to RNA processes (p<1.7e-08). Among the top ten most significant pathways, we also identified the pathway “Cellular response to stimuli” (p=4.1e-06) enriched, but none of the identified pathways was associated with pro-inflammatory immune functions. Considering all pathways, we identified three of them to be enriched within the Immune System including Neutrophil degranulation (p=8.1e-03), Antiviral mechanism by IFN-stimulated genes (p=1.65e-02), and Cross-presentation of soluble exogenous antigens (p=1.65e-02). However, the statistical significance of the enrichment for these pro-inflammatory pathways was significantly lower in comparison to RNA-associated pathways. No enrichment of Immune System-associated pathways was observed for proteins that were exclusively identified in MAIT cells after Ac-6-FP stimulation or under unstimulated conditions. In THP-1 cells, the number of proteins that were exclusively expressed in each condition was very similar and ranged from ten to 12 proteins. Proteins that were specifically translated in THP-1 cells following co-culture with 5-OP-RU-stimulated MAIT cells included the Guanylate-binding protein 3 (GBP3), which is known to exhibit antiviral functions during influenza infections (55). However, Reactome analysis did not reveal a relevant enrichment of inflammatory pathways in any of the conditions for THP-1 cells.

In the next step, we focused on protein translations that were not exclusive for a specific stimulation condition but showed robust MR1-ligand-dependent regulations in the bicellular system (log_2_FC>1; p-value<0.05) (**Table 2**; **Figure 3A** and **Supplementary Table 1**, **2**).

In 5-OP-RU-stimulated MAIT cells, 690 proteins were differentially upregulated whereas only six proteins were downregulated. In line with Reactome-based enrichment analysis, the most upregulated proteins included Interferon-induced proteins such as ISG15, MX1, GBP2, or OAS2, the NFκB-associated proteins NFKB1 and NFKB2 as well as pro-inflammatory proteins MAP2K3 or NR4A3. In contrast, we could not detect an upregulation of any of these pro-inflammatory candidates after Ac-6-FP stimulation (**Figure 3A**). While the increase in protein biosynthesis in THP-1 cells was not as pronounced as in MAIT cells, we still identified 47 proteins that were differentially upregulated after 5-OP-RU stimulation and 18 proteins that were downregulated. In contrast, no difference in protein up- or downregulation was observed following Ac-6-FP stimulation.

**Figure 4:**
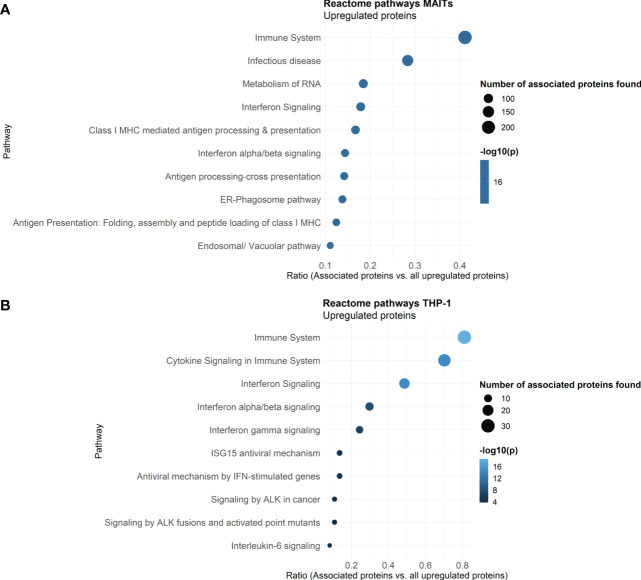
Reactome Pathway analysis of 5-OP-RU stimulated MAIT and THP-1 cells. Pathway analysis of translatome data. Differentially upregulated proteins (except low-confident proteins) from MAIT cells **(A)** and THP-1 cells **(B)** stimulated in the bicellular system were analyzed. The top ten enriched pathways sorted by p-values are shown. Dot size represents the number of proteins upregulated in the pathway, color represents the statistical significance of the pathway upregulation, x-axis represents the ratio of upregulated proteins in this pathway in comparison to all analyzed upregulated proteins, y-axis shows the Pathway name. P-values were determined by the Reactome database using hypergeometric distribution and corrected for false discovery rate using the Benjamini-Hochberg method.

To further classify the 5-OP-RU-regulated protein translations in MAIT cells, we again analyzed those at the level of pathways using the Reactome database. A look at the ten most enriched signaling pathways (sorted by p-value) revealed that after 5-OP-RU stimulation, MAIT cell protein translation supports Interferon signaling and Infectious diseases, highlighting their pro-inflammatory profile (**Figure 4A**). Additionally, pathways related to Antigen Processing and Presentation were enriched as well as Metabolism of RNA, which is in accordance with the group of exclusively identified protein translations described before.

In contrast, no pathways were significantly enriched following Ac-6-FP stimulation of MAIT cells. From the 12 upregulated proteins, five proteins (namely PUS7L, GSK3B, SNYVN1, SCAMP2, APRT) can be classified as highly specific for Ac-6-FP stimulated MAIT cells, i.e. they were not regulated after 5-OP-RU stimulation and were also not identified in AHA-free controls. Among them, GSK3B is a negative regulator of the duration of T cell responses and reduces the expression of Interleukin-2 (56). In the same line, candidates that were downregulated following Ac-6-FP stimulation also supported an anti-inflammatory function. These candidates included, for example, the metalloprotease ADAM10, which is expressed on activated T cells to form a mature immunological synapse 57), or the macrophage migration inhibitory factor (MIF) which is involved in the innate immune response to bacterial pathogens (58). Indeed, MIF was oppositely upregulated in MAIT cells in response to 5-OP-RU, suggesting an important role of MIF in regulating the phenotype of MAIT cells.

Next, we focused on the translatome response of THP-1 cells co-cultured with MAIT cells (**Figure 3B**). Similar to MAIT cells, translation of Interferon-stimulated proteins (GBP2, IFIT5, ISG15, MX1, STAT1, STAT3) and pro-inflammatory proteins such as NFKB2 were highly upregulated in THP-1 cells following co-culture with 5-OP-RU stimulated MAIT cells. However, the strongest increase in translation was found for APC-specific proteins such as TAP1. Proteins of the TAP family are involved in MHC class I antigen presentation and are known to be upregulated following Interferon-induction (59).

Reactome analyses further revealed that translation of THP-1 cells co-cultured with 5-OP-RU-activated MAIT cells supports pathways related to Immune system, Cytokine Signaling in Immune System, Interferon Signaling (e.g. Interferon (-α, -β, -γ), and Interleukin-6) as well as Antiviral mechanisms by IFN-stimulated genes or ISG15 (**Figure 4B**). In addition, Signaling by ALK was enriched among the top then pathways. Anaplastic lymphoma kinase (ALK) is activated in cancer and initiates downstream STAT3 signaling (60). Again, no pro-inflammatory pathways were enriched following Ac-6-FP stimulation.

Finally, we performed additional translatome experiments to define monocellular responses toward MR1-ligands (**Supplementary Tables 3**, **4**). In particular, we were interested in MR1-dependent self-reactivity of MAIT cells (**Figure 1B**) as well as in the sole effect of MR1-ligands on THP-1 cells. Interestingly, the comparison of data obtained in the bicellular vs monocellular system identified the importance of inter-cellular communication for regulating translatomes. Unstimulated MAIT cells cultured in the absence of THP-1 cells gave rise to the translation of 264-467 proteins compared to 933-1230 protein translations in the presence of APCs, indicating the dependency of MAIT cell protein translations on the presence of THP-1 cells (**Supplementary Figure S6E**). In contrast, the number of identified protein translations in unstimulated THP-1 cells was found to be less dependent on the presence of MAIT cells. For unstimulated THP-1 cells cultivated in the absence of MAIT cells, we observed 2631-2717 protein translations compared to 2893-3468 protein translations in the bicellular system (**Supplementary Figures S6B**, **F**). We further compared the overlap of protein translations in the mono- and bicellular systems. 1081 MAIT/2144 THP-1 protein translations were simultaneously detected in the mono- and bicellular system while 561 MAIT/802 THP-1 translations were exclusively found in monocellular-stimulated cells and 1182 MAIT/110 THP-1 proteins were only identified in bicellular-stimulated cells (**Supplementary Figure S8A**). Interestingly, 108 proteins were upregulated in MAIT cells stimulated with 5-OP-RU in the absence of APCs (**Supplementary Table S9**; **Supplementary Figures S8B**, **C**, top). Thus, to a certain extent, 5-OP-RU can directly induce protein translations in MAIT cells even in the absence of APCs but the induced translational response is far lower compared to the activation in the presence of THP-1 cells where 690 proteins were found to be upregulated. Pathway analyses of auto-stimulated MAIT cells revealed that RNA-associated processes were more relevant than pro-inflammatory (e.g. Interferon) responses which were strongly enriched in the MAIT cell translatome upon bicellular activation (**Supplementary Figure S7B**). Among the top 25 pathways, only three inflammatory pathways were enriched in the monocellular system: Influenza Viral RNA Transcription and Replication (5.3e-04), Cytokine Signaling in Immune System (p=2.0e-04), and Influenza Infection (p=0.013). Translations of pro-inflammatory MIF or NFKB2, which were 5-OP-RU induced in the bicellular system were detected but were not found to be upregulated in MAIT cells stimulated in the absence of APCs. In addition, the translations of other top MAIT cell candidates identified before in the more physiological bicellular system such as MX1, ISG15, OAS2, or OASL were not identified. Upon monocellular stimulation of MAIT cells with Ac-6-FP, inflammation-associated candidates such as GSK3B, MIF, or ADAM10 that were regulated during stimulation in the bicellular system were not detected or translation was not altered (**Supplementary Figures S8B, C**, top). 31 proteins were significantly upregulated but not related to Immune system processes. The three most enriched Reactome pathways were associated with Metabolism (Triglyceride catabolism), Metabolism of RNA (Formation of editosomes by ADAR proteins) as well as Transcription (Transcriptional Regulation of E2F6).

In contrast to the monocellular MAIT cell stimulation, only 13 proteins were upregulated after the loading of THP-1 cells with 5-OP-RU (**Supplementary Figures 8B, C**, bottom; **Supplementary Table S9**). These protein translations from the monocellular system were not associated with any pro-inflammatory processes. Some of the ISGs (including ISG15, MX1, STAT1) but also TAP-related proteins and GBP proteins that were highly upregulated in the bicellular system following 5-OP-RU stimulation in THP-1 cells were also detected in the monocellular system, but importantly, not differentially regulated in comparison to unstimulated samples. Thus, our data suggest that the basal proteome repertoire of THP-1 cells already comprises all proteins required for MR1 trafficking, such that no new protein translation is required, explaining why the response is not visible by our method.

In summary, analyzing the translatome of MAIT and THP-1 cells after 5-OP-RU stimulation revealed a strong pro-inflammatory and type I Interferon-driven profile in both cell types. Furthermore, protein translations are dependent on the intercellular crosstalk and MR1-activated MAIT cells can induce an inflammatory response of THP-1 monocytes.

### 5-OP-RU-activated MAIT cells drive THP-1 cells into an M1-like phenotype

**Figure 5:**
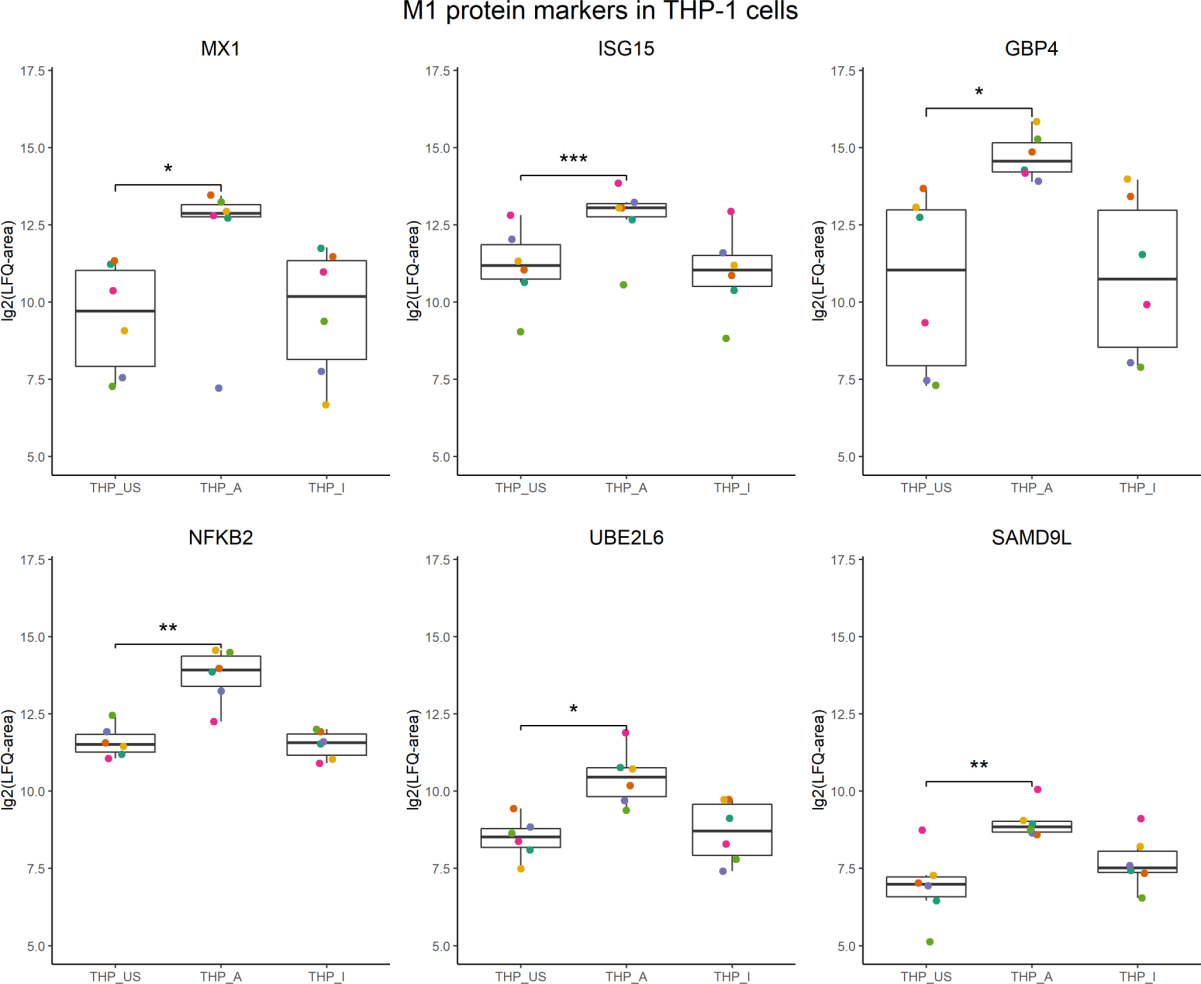
M1 protein candidates in the translatome of THP-1 cells co-cultured with 5-OP-RU-activated MAIT cells. M1 protein markers described in Huang et al, 2018 with an M1/M0 ratio ≥ 4 were compared with the translatome of THP-1 cells after 5-OP-RU stimulation with MAIT cells. Proteins shown were defined as M1 markers, and differentially upregulated (p<0.05, log_2_(FC)>1, except low-confident proteins) in the translatome. p*≤ 0.05; p**≤ 0.01; p***≤ 0.001.

In the presence of 5-OP-RU-activated MAIT cells, THP-1 cells increased translation of proteins associated with interferon signaling, including ISG15, STAT1, MX1, and GBP4. Interestingly, other proteins such as NKFB2, UBE2L6, and SAMD9L that were found as well upregulated in the translatome of THP-1 cells, have been described before as markers for M1 macrophages (61, **Figure 5**).

**Figure 6:**
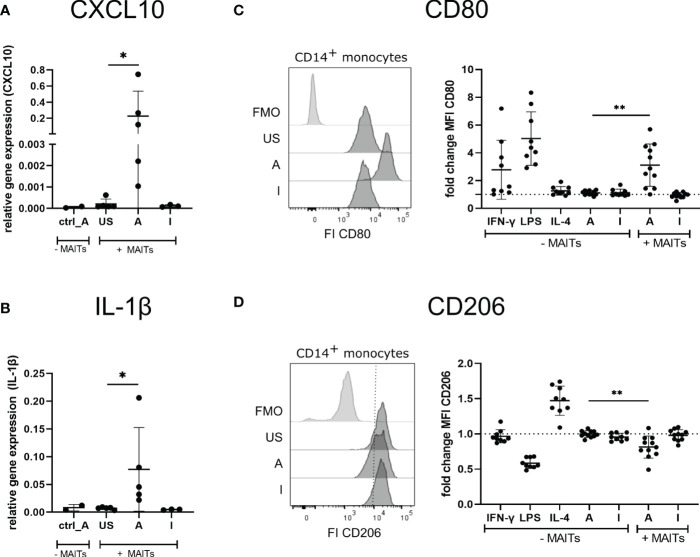
5-OP-RU-activated MAIT cells induce an M1-like phenotype of macrophages. **(A, B)** THP-1 cells were stimulated ± FACS-sorted MAIT for 20 hours with 50 ng/ml 5-OP-RU or Ac-6-FP. Cells were separated by FACS after stimulation. Total RNA was isolated and used for RT-qPCR. Relative gene expression in comparison to GAPDH is shown. Data from two independent experiments from five donors are shown. **(C, D)** FACS sorted CD14^+^ monocytes were differentiated into M0 phenotype with M-CSF for 8 days. M0 macrophages were paired with FACS-sorted MAIT cells of the same donor and stimulated for 20 hours with 50 ng/ml 5-OP-RU or Ac-6-FP. Surface expression of CD80 and CD206 on macrophages was determined by flow cytometry. **(C, D, left)** Representative histograms showing MFI of CD80 and CD206 on macrophages incubated in the presence of MAIT cells or showing Fluorescence minus one (FMO) staining. **(C, D, right)** Fold change in comparison to unstimulated macrophages (± MAIT cells) is shown. Data from four independent experiments from six to nine donors are summarized. **(A-D)** Asterisks indicate significant differences determined by Wilcoxon matched-pairs signed rank test; p* < 0.05; p** < 0.01. Horizontal lines indicate mean ± SD. US = unstimulated; A = 50 ng/ml 5-OP-RU; I = 50 ng/ml Ac-6-FP; FI = Fluorescence intensity.

Thus, we hypothesized that 5-OP-RU-activated MAIT cells may induce the polarization of THP-1 cells into an M1 phenotype. To test this hypothesis, we characterized gene expression of the prototypical M1 markers CXCL10 and IL-1ß, which because they are secreted are not suitable for cellular translatome analyses. Quantitative Real-time PCR (qRT-PCR) indeed showed upregulation of *CXCL10* and *IL-1*β in THP-1 cells in presence of 5-OP-RU-activated MAIT cells (**Figures 6A, B**). In contrast, we neither observed M1 marker induction following Ac-6-FP-mediated MAIT cell stimulation nor in 5-OP-RU-loaded THP-1 cells in the absence of MAIT cells, suggesting that activated MAIT cells are crucial to mediate M1 polarization in THP-1 cells. Interestingly, donor variations were also distinctive in THP-1 cells, indicating that the responsiveness of MAIT cells from different donors is strongly influencing the polarization.

Next, we further validated the MAIT cell-dependent induction of an M1 macrophage phenotype using primary MAIT cells and CD14^+^ monocytes of the same donors. For this purpose, primary CD14^+^ monocytes were isolated from PBMCs and differentiated into M0 macrophages during an eight days culture in the presence of Macrophage colony-stimulating factor (M-CSF). M0 macrophages were then co-cultured with MAIT cells of the corresponding donors in the presence of 5-OP-RU or Ac-6-FP and subsequently, the macrophage phenotype was assessed by flow cytometry (**Supplementary Figure S10A**). As expected, M0 macrophages stimulated with IFN-γ or Lipopolysaccharide (LPS) in the absence of MAIT cells developed an M1 phenotype as indicated by the elevated surface expression of the M1 marker CD80 (**Figure 6C**). In contrast, Interleukin-4 (IL-4), 5-OP-RU, or Ac-6-FP stimulation in absence of MAIT cells failed to induce M1 polarization of M0 macrophages. Strikingly though, CD80 surface expression was significantly increased following 5-OP-RU stimulation in the presence of MAIT cells, while Ac-6-FP stimulation in presence of MAIT cells showed no effect. Analyzing the surface expression of the M2 marker CD206, we observed a 1.5-fold increase in presence of Interleukin-(IL-)4, which is known to induce an M2 phenotype (**Figure 6D**). In contrast, LPS decreased CD206 expression, while IFN-γ treatment showed almost no effect on CD206 expression. In line with our previous data, CD206 expression on macrophages was significantly decreased following co-culture with 5-OP-RU-stimulated MAIT cells, while this effect was gone in the absence of MAIT cells. As expected, the surface expression of CD206 was not affected by Ac-6-FP stimulation, irrespective of whether MAIT cells were present or not. Interestingly, we observed the highest CD80 expression of macrophages in those donors that also had the highest CD69 expression on MAIT cells (**Supplementary Figure S10B**). Thus, we speculated that these MAIT cells might also secrete the most IFN-γ, resulting in an efficient M1 polarization of the corresponding macrophages. However, IFN-γ in the supernatant did not correlate with CD69 or CD80 expression.

In conclusion, the combination of translatome, gene expression, and flow cytometry data consistently revealed the capability of 5-OP-RU-activated MAIT cells to support M1 polarization of macrophages.

### 5-OP-RU-activated MAIT cells induce an antiviral state in THP-1 cells

**Figure 7:**
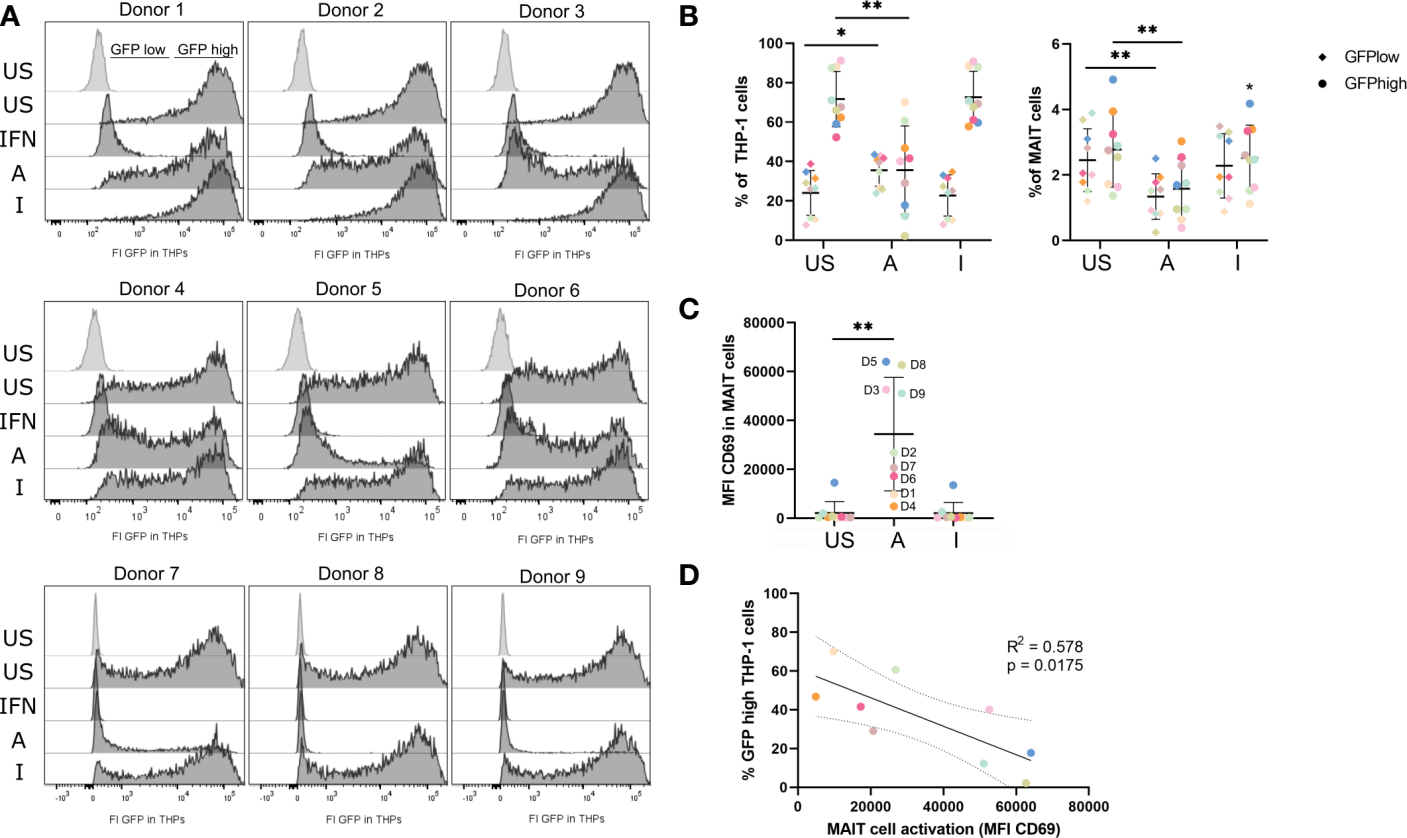
5-OP-RU-activated MAIT cells diminish intracellular viral levels in THP-1 cells. MAIT and THP-1 cells were stimulated for 20 hours with 50 ng/ml 5-OP-RU or Ac-6-FP. Subsequently, pre-stimulated cells were infected with VSV-eGFP (MOI 5) for 6 hours. Intracellular viral levels were quantified by measuring the GFP fluorescence by flow cytometry. **(A)** Histograms of GFP fluorescence of uninfected (light grey) and infected THP-1 cells (dark grey) are shown after pre-incubation with different MAIT cell donors. **(B)** Statistical evaluation of intracellular viral levels in THP-1 cells (left) and MAIT cells (right). **(C)** MAIT cell activation was determined by CD69 expression after 20 hours (D = Donor). **(D)** Correlation of MAIT cell activation and intracellular viral levels in THP-1 cells per donor. The solid line shows a linear correlation with a 95% confidence interval (dotted lines). R square (R^2^) of linear regression and p-value for the scope to be non-zero are given. (**C+D**) Each dot representing an individual donor is shown in a different color corresponding to the color in **(B)**. Data from three independent experiments from nine donors are shown. Asterisks indicate significant differences determined by Wilcoxon matched-pairs signed rank test; p* < 0.05; p** < 0.01. Horizontal lines indicate mean ± SD. US = unstimulated; A = 50 ng/ml 5-OP-RU; I = 50 ng/ml Ac-6-FP; IFN = Interferon αB/D; FI = Fluorescence intensity.

Since M1-polarized macrophages can exert critical roles in antiviral immunity and the translatome of THP-1 indicated up-regulation of antiviral pathways (**Figure 4B**) after co-culture with 5-OP-RU-stimulated MAIT cells, we wondered whether activated MAIT cells might induce an antiviral phenotype in THP-1 cells. Thus, we next analyzed the antiviral response in THP-1 cells after their co-culture with MAIT cells stimulated with MR1-ligands. For this purpose, we pre-incubated MAIT and THP-1 cells for 20 hours in the presence of either 5-OP-RU, Ac-6-FP, or a MOCK control before infecting them with vesicular stomatitis virus expressing the green fluorescent protein (VSV-eGFP) for six hours at MOI5. Intracellular viral levels were determined by flow cytometry, measuring the GFP fluorescence in live THP-1 and live MAIT cells. As expected, unstimulated THP-1 cells had high intracellular viral levels of VSV-eGFP, resulting on average in 71.7% GFP^high^ and 23.7% GFP^low^ THP-1 cells (**Figure 7A, B**, left). As a positive control for an antiviral state of THP-1 cells, cells were pre-stimulated for 20 hours with Interferon-αB/D. Here, the intracellular viral levels were completely suppressed, while the viral levels in THP-1 cells loaded with the MR1-ligands in the absence of MAIT cells were not altered (**Figure 7A**; **Supplementary Figure S11A**). Strikingly, we detected a significantly decreased GFP signal in THP-1 cells following their co-cultivation with MAIT cells in the presence of 5-OP-RU, suggesting that 5-OP-RU-activated MAIT cells are capable to induce an antiviral phenotype in THP-1 cells. On average, 35% fewer THP-1 cells were GFP^high^ in comparison to unstimulated cells and the frequency of GFP^low^ cells increased by 12% (**Figured 7A, B**, left). While a decrease of intracellular viral levels was observed for all nine donors, the strength of inhibition significantly correlated with the activation status of the MAIT cells as defined by CD69 expression. MAIT cells from donors exhibiting higher CD69 expression following stimulation induced a more pronounced antiviral phenotype in THP-1 cells than donors with a lower activation status (**Figures 7C, D**). Furthermore, the degree of inhibition of intracellular viral levels in THP-1 cells elevated with increasing doses of 5-OP-RU. At 5 ng/ml 5-OP-RU, we also detected significantly more GFP^low^ and significantly fewer GFP^high^ THP-1 cells (**Supplementary Figure S11B–D**). As expected, the effect was less pronounced correlating again with the activation status of MAIT cells in the system. In comparison to THP-1 cells, for which we observed on average 71.7% GFP^high^ and 23.7% GFP^low^ cells following infection with VSV-eGFP in the unstimulated condition, we observed only 2.8% GFP^high^ and 2.4% GFP^low^ infection of MAIT cells. Nevertheless, intracellular viral levels were significantly diminished in MAIT cells activated with 50 ng/ml 5-OP-RU (**Figure 7B**, right). The frequency of both GFP^high^/GFP^low^ MAIT cells was on average reduced to 1.6%/1.3%. However, intracellular viral levels were not significantly altered in MAIT cells stimulated with 5 ng/ml 5-OP-RU (**Supplementary Figures S11E, F**).

Blocking of MR1 before stimulation with 5-OP-RU significantly increased intracellular viral levels in both MAIT and THP-1 cells, underlining the MR1-dependency of the antiviral mechanism (**Supplementary Figures S11G, H**). In addition, we analyzed intracellular viral levels upon suppression of the IFN-γ receptor (IFNGR1, CD119). In contrast to MR1 inhibition, blockage of IFNGR1 did not significantly upregulate intracellular viral levels in THP-1 cells. Instead, the amount of GFP^high^ THP-1 cells was comparable and not significantly altered in comparison to 5-OP-RU or 5-OP-RU plus Isotype control-treated cells (**Supplementary Figure S11I**). Similarly, intracellular viral levels in MAIT cells were not significantly altered after IFNGR1 inhibition in comparison to the Isotype control (**Supplementary Figure S11J**). Finally, we analyzed whether 5-OP-RU-activated MAIT cells can also induce an antiviral phenotype in naïve monocytes that were not involved in MAIT cell activation before. For this purpose, we pre-stimulated MAIT cells using plate-bound MR1-5-OP-RU- or MR1-Ac-6-FP-monomers before co-culturing them with THP-1 cells for 20 hours and infecting them with VSV-eGFP. Indeed, MR1-5-OP-RU-activated MAIT cells were able to induce an antiviral state in naïve THP-1 cells, similar to MAIT cells activated by THP-1 cells as previously described (**Supplementary Figures S11K–M**).

In summary, intracellular viral levels were significantly reduced in THP-1 following co-culture in the presence of 5-OP-RU-activated MAIT cells indicating that activated MAIT cells are capable of inducing an antiviral state in APCs.

## Discussion

Omic technologies have been successfully applied for the characterization of virtually all immune cell subsets including MAIT cells. In particular, transcriptomics has provided genome-wide information on gene expression following immune cell activation (16, 20, 21). However, information on differential protein translation in MAIT cells responding to certain stimuli is rare, probably because this requires the use of specific labeling technologies. For example, stable isotope labeling by amino acids (SILAC) is well established in quantitative proteomics and used for translation analyses, but such events have to be analyzed as part of whole proteomes limiting the throughput and sensitivity of such approaches (62).

On the contrary, BONCAT facilitates the selective examination of translational dynamics by combining labeling and “Click”-chemistry, to enrich specifically those proteins for the MS analysis that were newly translated. Due to its selectivity, BONCAT was successfully adapted for cell-specific *in vivo* proteomics (63) and the recognition of translationally active bacteria in the microbiota (64), but is equally suitable for the analysis of immune responses. With respect to immune responses, BONCAT was so far successfully utilized to detect early changes in protein expression in primary resting T cells subjected to activation stimuli (65) and to study secretomes of lipopolysaccharide-activated macrophages (66).

In this study, we have used BONCAT proteomics to complement information on MAIT cell immune responses towards MR1-ligands and analyzed protein translations quantitatively by LC-MS/MS. In addition, our study characterizes the immune response of THP-1 cells upon uptake of MR1 ligands and following intercellular crosstalk with activated MAIT cells. This allowed the characterization of in total 2263 newly translated proteins in MAIT cells and 3254 in THP-1 cells. Further data interpretation in this study was restricted to protein translations that were detectable and regulated before and after MR1-ligand stimulations, although exclusively identified or non-AHA-specific (low-confident) proteins also indicate interesting candidates (**Supplementary Table 5** and see below). In particular, proteins identified exclusively in activated MAIT or THP-1 cells potentially represent interesting candidates that are newly translated in response to MR1-ligands. However, the missing identification of such candidates is not sufficient evidence for their non-translation in unstimulated conditions and requires further validation. Thus, prospective studies may combine *in vivo* BONCAT and additional *ex vivo* labeling strategies such as TMT or iTRAQ allowing to confirm exclusive protein translation. Since we here used label-free quantification, we focussed on differentially translated proteins that were robustly detected in all conditions. One of the limitations of this study is that we cannot differentiate between TCR- and cytokine-induced translations after 20 hours. The major focus of this manuscript was not the dissection between cytokine-dependent and contact-dependent responses but to analyze the overall response towards MR1-ligands. Although 5-OP-RU did not increase the production of inflammatory cytokines by THP-1 cells in absence of MAIT cells, MR1-dependently activated MAIT cells can indeed influence THP-1 cells which might induce cytokine production (e.g. IL12 or IL15). IL12 and IL15 are known to promote antigen-dependent effector functions in MAIT cells but also to induce MAIT cell activation and cytolytic molecule expression in MAIT cells TCR-independently (67, 68). Another limitation of this study is that it focuses on cellular compartments as exemplified by the detection of CD69 which we found upregulated following 5-OP-RU stimulation. In contrast, prototypic secreted effector functions such as IFN-γ and Granzymes could not be covered and would require separate secretome analyses.

In comparison to transcriptomics, translatomics is complementing but not fully covering previous results. As expected, translatomes confirm the 5-OP-RU-induced protein translation of several type I IFN signaling proteins in activated MAIT cells, including IFIT1, ISG15, IFI44L, and IFIT3, which was suggested previously by single-cell transcriptomics (69). Furthermore, we found similar pathways enriched in response to TCR-stimulation as previously identified by transcriptomics studies including “Inflammatory response” and “Cytokine-mediated signaling pathway” (19). However, we were not able to identify a complete tissue repair signature of MAIT cells as described by transcriptomics of TCR-dependently activated MAIT cells (20, 21). The reason for this might be that 5-OP-RU-induced tissue repair factors are either secreted shortly after translation or possibly also the timing of their investigation. Previously, wound healing was examined using MAIT cell supernatants after 72 hours of stimulation with 5-OP-RU (21), whereas we stimulated MAIT cells for only 20 hours before translatome analyses. Thus, translatomics of this study provides only information on some early tissue-affecting factors, such as NR4A3. NR4A3 (or NOR-1) is a nuclear receptor whose 5-OP-RU-induced upregulation in MAIT cells was shown before at the gene expression level (70) and was now confirmed at the protein level. NOR-1 is involved in the regulation of inflammation and vascular remodeling and is induced by VEGFA, one of the key candidates described in the tissue repair profile of MAIT cells (21, 71). More detailed profiling of secretomes would be of interest to complement information on MAIT cell effector functions and click-based approaches such as BONCAT can detect those factors with high selectivity.

One of the most significant findings in this study was the environment-dependent global protein translation of MAIT cells. MAIT cells cultivated in the absence of any other cells exhibited a base level of only 463 protein translations, which increased in the presence of THP-1 cells (1013 translations) and was further inducible by 5-OP-RU (1708 translations). In contrast, the global protein translation of THP-1 cells was not as much increased as observed for MAIT cells. On the one hand, this could indicate that the signaling is mostly influencing the MAIT cells. On the other hand, we can also speculate that we would have observed a greater difference in the overall translation response of monocytes if we had used primary APCs. Activation of primary cells results in a physiological response, whereas cell lines exhibit basal activities and lower response dynamics. This is in line with our translatome data from MAIT and THP-1 cells, which showed notable induction of protein translation in the case of MAIT cells only. Indisputably, our results raised the question of how MAIT cell translation is controlled. In line with previous studies (72), we observed an *in vitro* auto-presentation of 5-OP-RU by MAIT cells which was, however, less effective to activate MAIT cells and enhance translation than the presentation of 5-OP-RU by professional antigen-presenting THP-1 cells. Besides, we observed the formation of MAIT-THP-1-conjugates also with Ac-6-FP and in absence of MR1 ligands but detected polarization at the IS only in presence of 5-OP-RU. Thus, MAIT cell translation is most prominently increased following antigen-dependent IS formations with professional APCs. Nevertheless, we may speculate that the self-activation of MAIT cells plays a subordinate role *in vivo* because of the ubiquitous expression of MR1 in tissues. Translatomics also detected Programmed cell death 4 (PDCD4) which might be a regulator of protein translation in MAIT cells. PDCD4 is a translation inhibitor (73) and was downregulated in MAIT cells following 5-OP-RU-mediated stimulation by THP-1 cells (log_10_(p)=1.43; lg_2_(FC)= -0.95). Indeed, the downregulation of PDCD4 might further support the pro-inflammatory phenotype of MAIT cells since cytotoxic T lymphocytes deficient in PDCD4 were described previously to express an increased amount of effector molecules such as IFN-γ (74). Of note, our analyses did not clarify whether co-receptor usage is influencing MAIT-THP-1-conjugate formation. By using newly developed MR1 tetramers mutated at the CD8 binding site it was recently (2022) shown by Souter et al. that CD8αα and CD8αβ enhance MR1 binding and cytokine production by MAIT cells. Thus, coreceptor usage could also influence conjugate formation and the subsequent translatome response in response to 5-OP-RU or Ac-6-FP, and together with results from our study, one may hypothesize that only CD8^+^ MAIT cells can form conjugates in the absence of 5-OP-RU.

In the same line, the results of this study emphasize that some effector functions of MAIT cells can only be mediated in conjunction with other cells. Here, the translatome of THP-1 suggested that 5-OP-RU-activated MAIT cells polarize THP-1 cells and primary macrophages into an M1 phenotype. Macrophage polarization was analyzed by proteomics of THP-1 cells before and M1 and M2 markers were described following LC-MSMS measurement (61). Several M1 markers that were identified by Huang et al. were also identified in our translatome study whereas none of the M2 markers was detected. Most importantly, these M1 markers were all either upregulated or not regulated compared with unstimulated samples in the translatome data, whereas none of them was downregulated. Likewise, we were able to confirm the M1 polarization also by using RT-qPCR and flow cytometry, strongly suggesting that 5-OP-RU-activated MAIT cells are able to induce an M1-like phenotype in macrophages. Notably, no other cell population or the contribution of an *in vivo* milieu was necessary to induce macrophage polarization. Thus, MAIT cell activation alone is sufficient to modulate macrophage phenotypes *in vitro* and could enable them to indirectly control infections. We hypothesized that IFN-γ secretion by MAIT cells could be responsible to induce the M1 polarization. However, IFN-γ in the supernatants did not correlate with CD80 expression on macrophages and the underlying mechanism of the MAIT cell-induced M1 polarization remains elusive.

MAIT cell-induced macrophage polarization was previously described in the context of two diseases. MAIT cells promote M2 polarization of macrophages in non-alcoholic fatty liver disease (NAFLD) by the production of regulatory cytokines (high production of IL-4, but reduced IFN-γ and TNF levels) (75). In contrast, MAIT cells in obesity induced M1 polarization of macrophages in an MR1-dependent manner, thereby promoting inflammation with high TNF production and metabolic dysfunction. On the one hand, *in vivo* treatment of obese mice with Ac-6-FP was able to decrease MAIT cell activation and M1 polarization, improving metabolic parameters (76). On the other hand, M1-polarized macrophages play essential roles in fighting against viral infections, e.g. by producing an oxidized environment and antiviral cytokines (77). Our data now confirm that MR1-activated MAIT cells from healthy individuals can induce M1 polarization and simultaneously promote the induction of an antiviral macrophage phenotype.

Of course, there is growing evidence for the relevance of MAIT cells in immunity to viral infections. MAIT cell activation was associated with disease severity for example in COVID-19 or hantavirus infections (5, 78), and conversely, they were shown to contribute to protection against lethal influenza infection *in vivo* and to suppress HIV-1 (7, 79). The induction of an antiviral phenotype by MR1-ligand-activated MAIT cells was examined in our study using vesicular-stomatitis virus expressing GFP (VSV-eGFP). However, the question of how the antiviral response is induced remains an open question. We speculate about four different mechanisms that potentially mediate the antiviral phenotype: First, type I IFNs are the most common cytokines that induce antiviral functions. They have been shown to increase 5-OP-RU-mediated MAIT cell activation (80) and stimulation of PBMCs with 5-OP-RU was recently described by single-cell transcriptional profiling to induce a strong type I IFN signaling signature in MAIT cells (69). However, to our knowledge, it is so far unclear whether MAIT cells can produce type I Interferons themselves. Secondly, we may speculate that IFN-γ is responsible for the induction of the antiviral phenotype. Although type I IFNs are the prototypic inducers of ISGs, IFN-γ also promotes the transcription of several antiviral genes by stimulating downstream signaling that activates the gamma-activated sequence (GAS) promotor (81). Since 5-OP-RU-activated MAIT can produce IFN-γ, this is a plausible mechanism of how the antiviral phenotype is induced in monocytes (21, 69). Third, granzymes, which are known to be produced during early activation of MAIT cells, can also contribute to the induction of antiviral phenotypes e.g. by cleaving host proteins required for viral replication (82). Nevertheless, the transmission of non-lytic granzyme-containing vesicles towards interacting APCs has not yet been described. Finally, the induction of the antiviral phenotype may be induced contact-dependently by synpase formation between activated MAIT and THP-1 cells. Interestingly, plaque assay revealed that supernatants collected after 20 hours of 5-OP-RU-stimulated MAIT and THP-1 cells were not able to induce an antiviral phenotype in VeroE6 cells (data not shown). This could indicate that the concentration of soluble factors in the supernatants was either too low or that soluble factors alone are not able to induce an antiviral phenotype. This hypothesis was further supported by blocking the IFN-γ receptor which did not increase intracellular viral levels. Using MAIT cell activation with plate-bound MR1-monomers, we were able to show that MAIT cells induce an antiviral phenotype also in naïve monocytes which were not involved in MR1-dependent activation before. Although we cannot clarify the contribution of soluble factors for the induction of an antiviral phenotype, we may finally speculate that the formation of an IS with activated MAIT cells is crucial for this mechanism.

Interestingly, apart from Interferon-stimulated proteins also other candidates such as CD70 substantiate the antiviral phenotype. Although CD70 was considered a low-confident protein translation (non-AHA-specific) due to its upregulation in both AHA- and non-AHA-samples, it is known to bind CD27 on T cells thereby promoting the generation of T cell immunity, particularly during antiviral responses (83, 84). Although CD27 was not detected in the translatome of MAIT cells in this study, MAIT cells are known to express CD27 on their surface and MAIT cell frequencies were even shown to be reduced in CD27/CD70 deficiency (85, 86). Thus, the CD70-CD27 axis might play an important co-stimulatory function in MAIT cell-mediated antiviral responses.

Moreover, there is also the question of the role of an MR1-mediated antiviral response. Previous studies suggest that MAIT cell activation in virus infections is primarily controlled cytokine-dependently, but our study now indicates the importance of MR1-ligands which occur in co-infection. In contrast, a virus-mediated MR1 regulation seems to be unlikely to date, since no mammalian and/or virally encoded MR1 ligand has been identified. Nevertheless, several herpesviruses such as HSV-1 and CMV were shown to disrupt MR1 surface expression, inhibiting MAIT-TCR-dependent activation (87) and recent studies indicated that MR1-restricted T cells might recognize distinct cell-derived antigens and describe MR1-dependent activation in the absence of microbial ligands 88, 89). Thus, regulation of antiviral responses by bacterial MR1-ligands during co-infections might play an important regulatory role, and evidence for MR1-dependent mechanisms in absence of bacterial infections is growing.

This translatome study sheds new light on inhibitory MR1-ligands. In parallel to 5-OP-RU, we analyzed the response of MAIT and THP-1 cells to the inhibitory ligand Ac-6-FP. In contrast to 5-OP-RU, the MR1-Ac-6-FP-complex is known to interact with the MAIT TCR with only low affinity (13). Therefore, we wondered whether the mode of action of Ac-6-FP is restricted to its MR1 binding on THP-1 cells or whether the MR1-Ac-6-FP complex is capable of inducing any kind of response with interacting MAIT cells. Therefore, we analyzed the IS formation of MAIT and THP-1 cells in the presence of both metabolites as well as the translatome following Ac-6-FP stimulation. Whereas we observed increased IS formation with MAIT and THP-1 cells in presence of 5-OP-RU and *E. coli*, Ac-6-FP was not impacting conjugation frequencies nor induced notable IS formations. However, Ac-6-FP-related translatomes revealed a small number of interesting regulated protein translations. For instance, we identified the downregulation of the Disintegrin and Metalloproteinase Domain-containing protein 10 (ADAM10) in the translatome of MAIT cells co-cultured with Ac-6-FP-loaded THP-1 cells. ADAM10 is localized at the central part of the supramolecular activation cluster (cSMAC) of the IS in TCR-engaged T cells and promotes the formation of a mature IS. Thus, decreased translation of ADAM10 restricts IS formation and may be part of an anergy-like phenotype of MAIT cells that was previously only described in response to superantigens due to exhaustion (90). This assumption is supported by the simultaneous upregulation of Glycogen synthase kinase-3 beta (GSK3B), which negatively regulates the duration of T cell responses and reduces the production of Interleukin-2 (IL-2, 56) as well as the downregulation of the Macrophage migration inhibitory factor (MIF), which is promoting the IL-2 production in T cells (58). Interestingly, MIF was differentially upregulated in 5-OP-RU-activated MAIT cells, suggesting an important role in the regulation of MAIT cell immunity. In conclusion, the characterization of the IS, as well as the differential expression of ADAM10, GSK3B, and MIF, indicate a distinct regulatory or anergy-like phenotype of MAIT cells in response to Ac-6-FP. This may resemble the anergic phenotype described for conventional T cells, which enter a state of anergy in which they are unresponsive to subsequent stimulation with the agonist peptide if they were stimulated with either antagonistic or partially agonistic peptides before (91). It remains unknown whether the induction of the anergic phenotype is dependent on MAIT TCR and MAIT co-receptor usage. Furthermore, future studies need to clarify whether the anergic phenotype of MAIT cells post Ac-6-FP exposure is sustained and whether it is influencing 5-OP-RU-dependent activation.

In summary, translatome analyses of MAIT cells from healthy individuals revealed pro-inflammatory and type I Interferon-driven profiles in response to the microbial-derived MR1-ligand 5-OP-RU in both MAIT and THP-1 cells while it suggests a distinct anergy-like profile of MAIT cells in presence of Ac-6-FP. With the applied translatome strategy we were able to show that 5-OP-RU-activated MAIT cells influence the phenotype of macrophages by inducing an M1 and antiviral phenotype. Thus, MR1-ligands contribute to antiviral responses, which need to be considered in co-infections. In this line, BONCAT proteomics can certainly help to examine MAIT cell immune responses in different infectious diseases or transgenic *in vivo* models. Together with transcriptomics, this will clarify regulatory mechanisms and will broaden knowledge on direct as well as indirect effector functions based on the cross-talk with antigen-presenting cells.

## Data availability statement

The datasets presented in this study can be found in online repositories. The names of the repository/repositories and accession number(s) can be found below: PXD039785, PXD039804, PXD039810, PXD039820 (PRIDE).

## Ethics statement

The studies involving human participants were reviewed and approved by Ethics Committee of Lower Saxony, Germany. The patients/participants provided their written informed consent to participate in this study.

## Author contributions

DB and LJ conceived and designed the research. JJ designed and performed the experiments and analyzed the data. AK provided the VSV-eGFP virus. FK designed and performed the statistical analyses. JJ, DB, and LJ wrote the manuscript. All authors contributed to the article and approved the submitted version.

## Supplementary material

Supplementary Table 1 Bicellular_MAIT.

Supplementary Table 2 Bicellular_THP-1.

Supplementary Table 3 Monocellular_MAIT.

Supplementary Table 4 Monocellular_THP-1.

Supplementary Table 5 Exclusively identified proteins per conditions.
