# Supplementary material for: Translatome analyses by bio-orthogonal non-canonical amino acid labeling reveal that MR1-activated MAIT cells induce an M1 phenotype and antiviral programming in antigen-presenting monocytes
Source: Front Immunol. 2023 Feb 16;14:1091837. doi: 10.3389/fimmu.2023.1091837 (PMC9977998; doi:10.3389/fimmu.2023.1091837)
Supplement: Supplementary file 6 [file DataSheet_1.docx]

**Supplementary Figures:** Translatome analyses by bio-orthogonal non-canonical amino acid labeling reveal that MR1-activated MAIT cells induce an M1 phenotype and antiviral programming in antigen-presenting monocytes


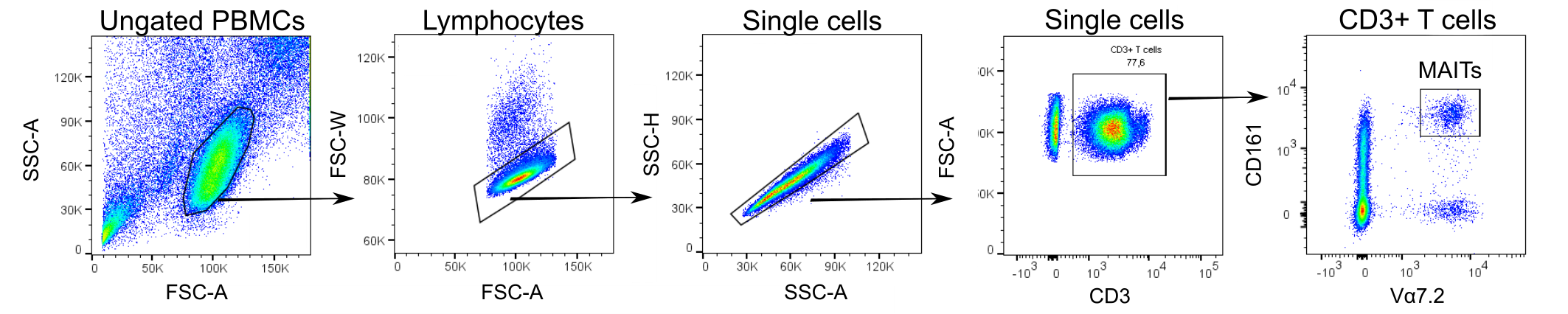


**Supplementary Figure S1: Gating strategy for Flow-cytometry associated cell sorting of human MAIT cells**. PBMCs were isolated from human blood. Lymphocytes were identified by forward scatter-area (FSC-A) and side scatter-area (SSC-A). Single cells were discriminated by forward scatter-area (FSC-A) and forward scatter-width (FSC-W) as well as side scatter-area (SSC-A) and side scatter-height (SSC-H). MAIT cells were identified as CD3^+^CD161^++^Vα7^+^ single-cell lymphocytes.


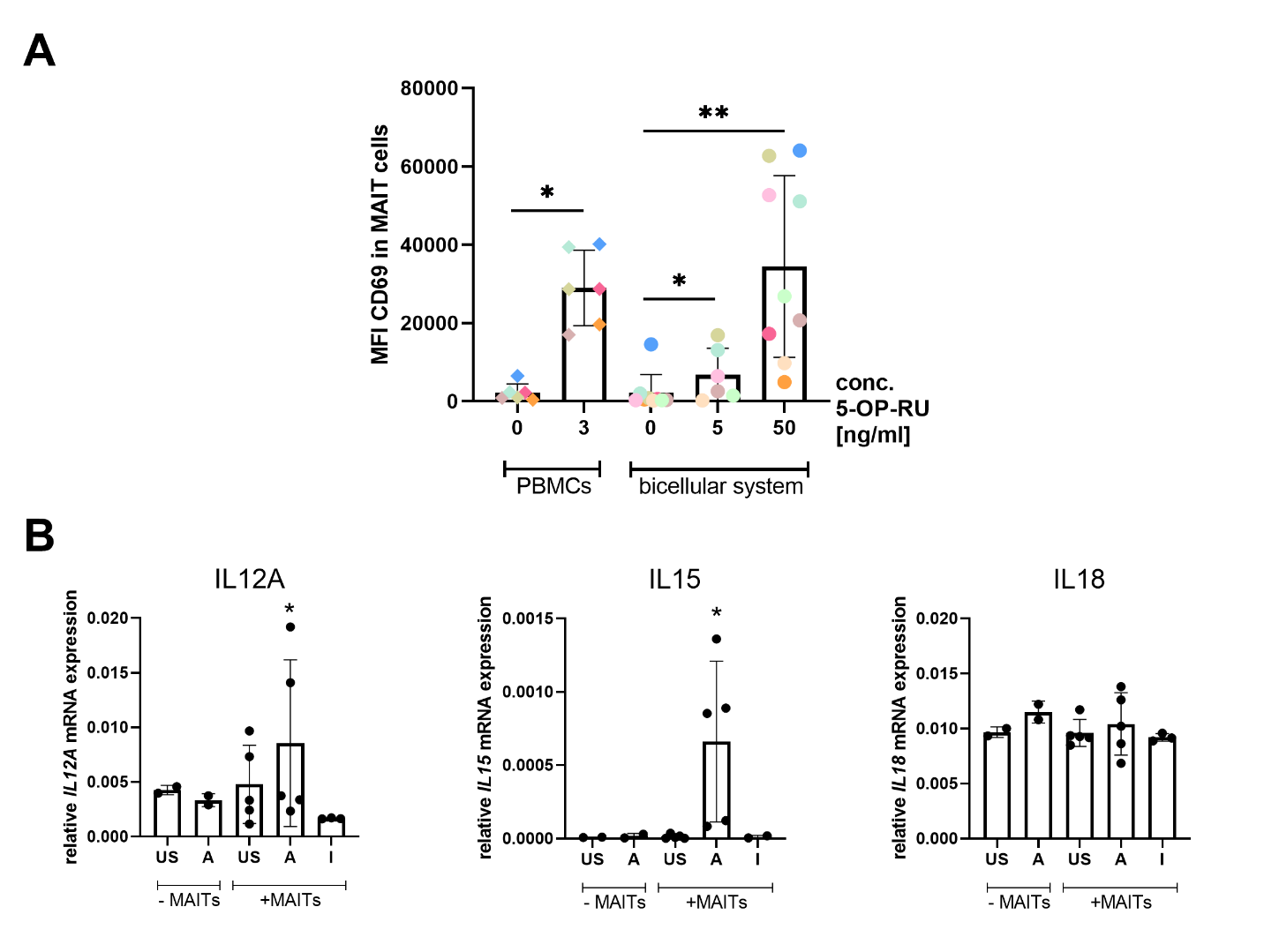


**Supplementary Figure S2: Establishment of the bicellular system.** **A**) 5-OP-RU-induced MAIT cell activation in PBMCs and the bicellular system. PBMCs or FACS-sorted MAIT cells plus THP-1 cells (bicellular system) were stimulated with indicated concentrations of 5-OP-RU. MAIT cell activation (MFI CD69) was assessed by flow cytometry after 20 hours. Same donors are shown in the same color. Data from two independent experiments from six to nine donors are shown. B) THP-1 cells were stimulated ±FACS-sorted MAIT for 20 hours with 50 ng/ml 5-OP-RU or Ac-6-FP. Cells were separated by FACS after stimulation. Total RNA was isolated and used for RT-qPCR. Relative gene expression in THP-1 cells in comparison to Rps9 is shown. Data from two independent experiments from five donors are shown. Asterisks indicate significant differences determined by Wilcoxon matched-pairs signed rank test; p*<0.05; p** < 0.01. Bars indicate mean ± SD. US = unstimulated; A = 50 ng/ml 5-OP-RU; I = 50 ng/ml Ac-6-FP.

**Supplementary Figure S3: Enrichment of newly synthesized proteins.** I) PBMCs were isolated from PBMCs of six healthy human donors (n=6). II+III) FACS-sorted MAIT and THP-1 cells were co-cultured in 1 mM AHA medium and were either treated with 50 ng/ml 5-OP-RU or Ac-6-FP or left untreated. IV) MAIT cells were separated from THP-1 cells by FACS after 20 hours stimulation. V) AHA-containing proteins were enriched by Click-it reaction where the reactive azide group of AHA is covalently coupled to small alkyne-bearing beads in a copper-catalyzed azide-alkyne reaction (Dieterich et al., 2007). VI+VII) Proteins were cleaved from beads by tryptic digest, peptides were purified by the SP3 method and subsequently measured by accurate LC-MS/MS. Parts of the Figure were created with BioRender.com.


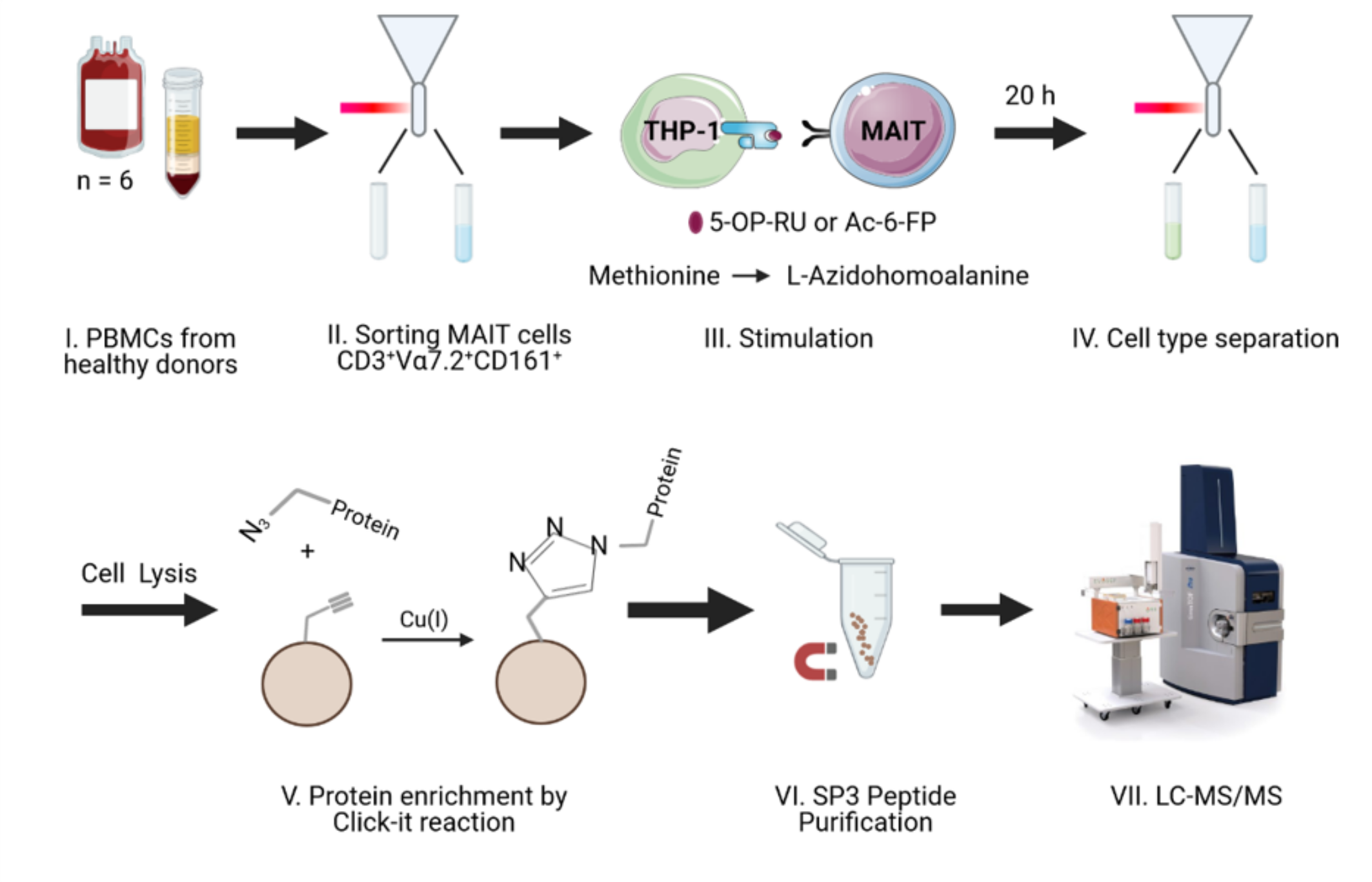


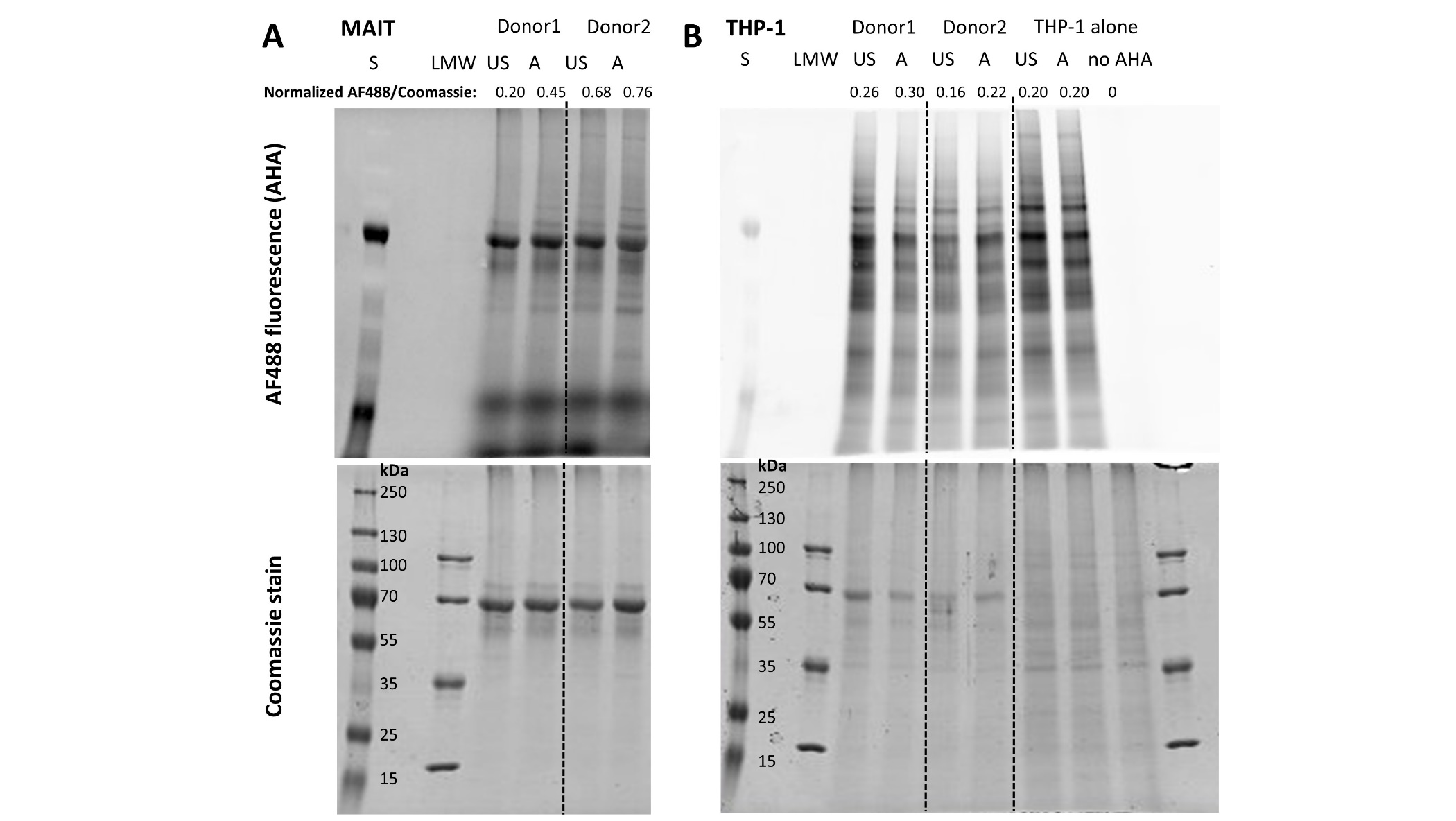


**Supplementary Figure S4: AHA incorporation efficiency determined by FUNCAT.** MAIT and THP-1 cells were separated by FACS after 20 hours stimulation (± 50 ng/ml 5-OP-RU) in 1 mM AHA medium. AHA in cell lysates was coupled to Alkyne-AF488 in a Click-reaction. Fluorescent lysates were separated on a 10% SDS gel before determining AF488 fluorescence. AF488 fluorescence and Coomassie stain of MAIT cells (A) and THP-1 cells (B) are shown. As controls, THP-1 cells were incubated without MAIT cells or AHA (B, lines THP-1 alone). S: PageRuler plus Stained protein Ladder; LMW: Low molecular weight protein marker; US = unstimulated; A = 50 ng/ml 5-OP-RU.


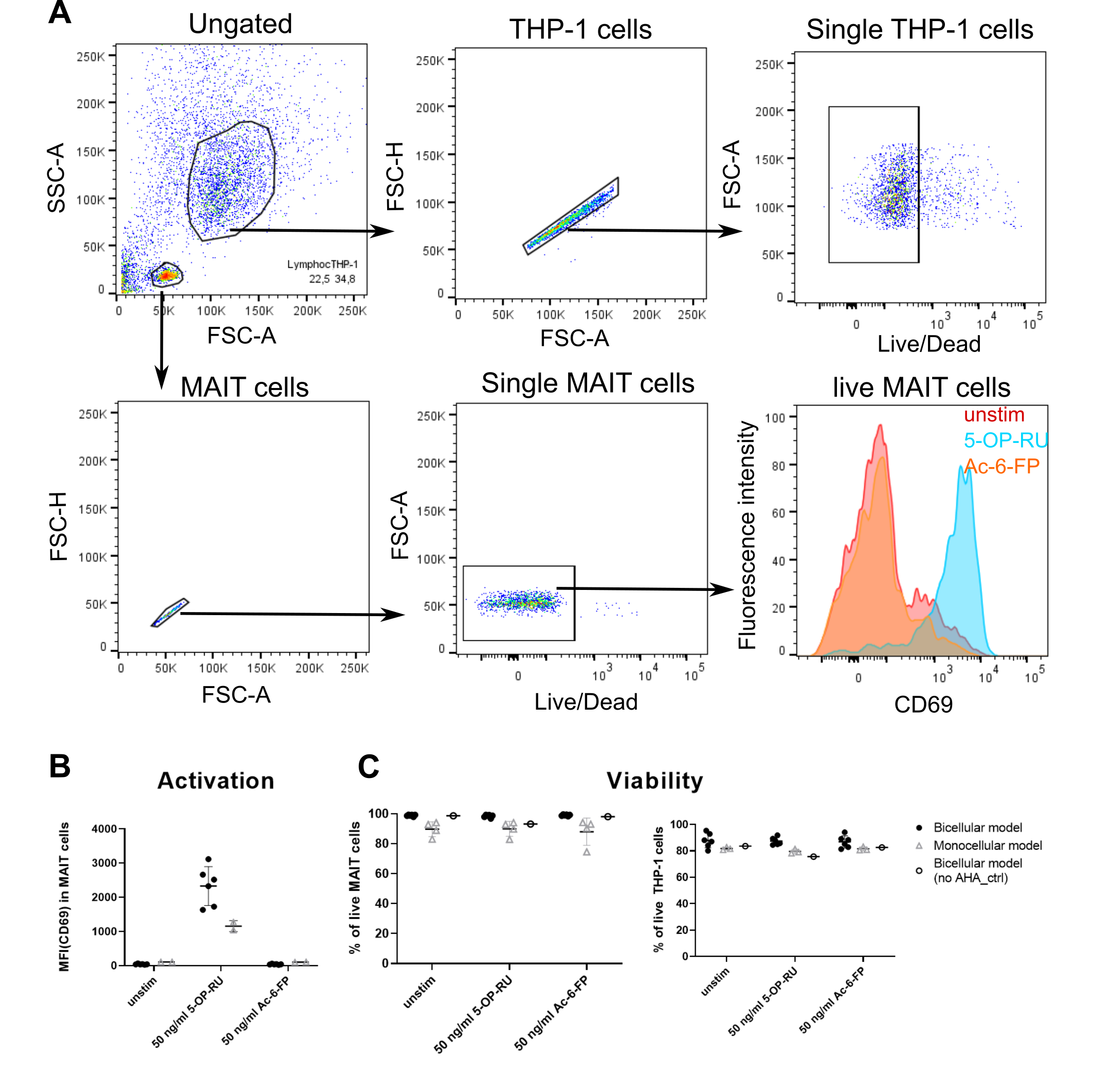


**Supplementary Figure S5: MAIT cell activation and cellular viability in translatome experiments. A**) Gating strategy to analyze MAIT cell activation (CD69) and MAIT/ THP-1 cell viability in the bicellular system by flow cytometry is shown. MAIT cell activation (**B**) and MAIT/THP-1 viability (**C**) of samples stimulated in L-Azidohomoalanine (AHA) medium for translatome analysis are shown after 20 hours. As Click-reaction enrichment control, one donor was stimulated without AHA (no AHA_ctrl). unstim = unstimulated

**
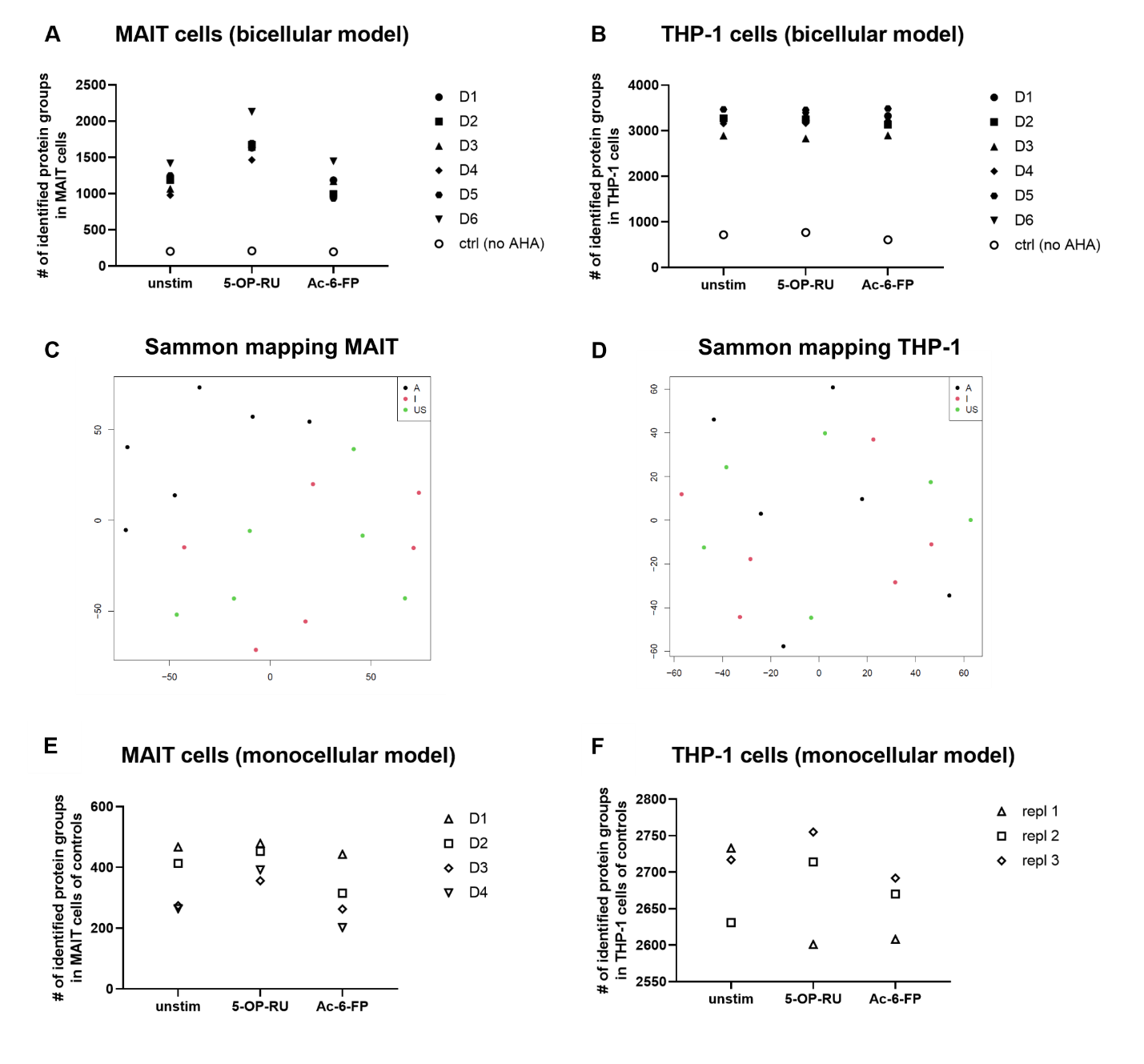
Supplementary Figure S6: Analysis of translatome raw data. A+B)** Identified protein groups given by PEAKS identification of MAIT cell donors after separation from THP-1 cells in the bicellular system (A) and THP-1 cells after separation from MAIT cells (B). Control (Ctrl) samples were stimulated in the bicellular system in normal medium without L-Azidohomoalanine (AHA). **C+D**) Sammon mappings of the translatome of MAIT (C) and THP-1 (D) cells were performed before imputing missing values for further data analysis. **E**) PEAKS protein identifications in MAIT cells stimulated without THP-1 cells, **F**) THP-1 cell stimulated without MAIT cells. repl = replicate; D=Donor; ctrl=control


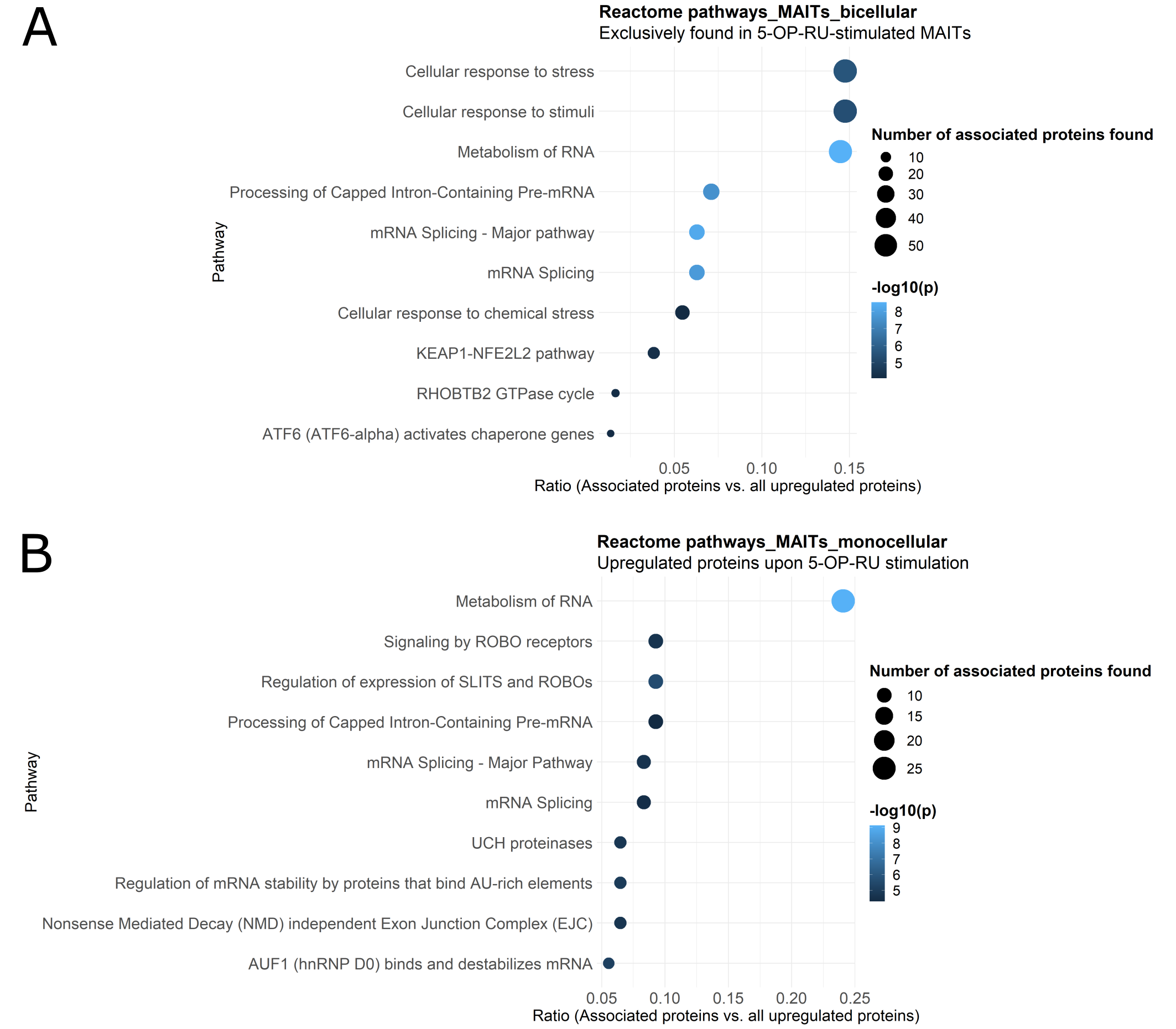


**Supplementary Figure S7:** **Reactome Pathway analysis of 5-OP-RU stimulated MAIT cells.** Pathway analysis of translatome data. (A) MAIT cell proteins that were newly synthesized exclusively in response to stimulation with 5-OP-RU-loaded THP-1 cells were analyzed. (B) Differentially upregulated MAIT cell proteins after monocellular 5-OP-RU stimulation were analyzed. (A+B) The top ten enriched pathways sorted by p-values are shown. Color represents the statistical significance of the pathway upregulation, dot size represents the number of proteins upregulated in the pathway, y-axis shows the pathway names, x-axis represents the ratio of upregulated proteins in this pathway in comparison to all analyzed proteins. P-values were determined by the Reactome database using hypergeometric distribution and corrected for false discovery rate using the Benjamini-Hochberg method.

**
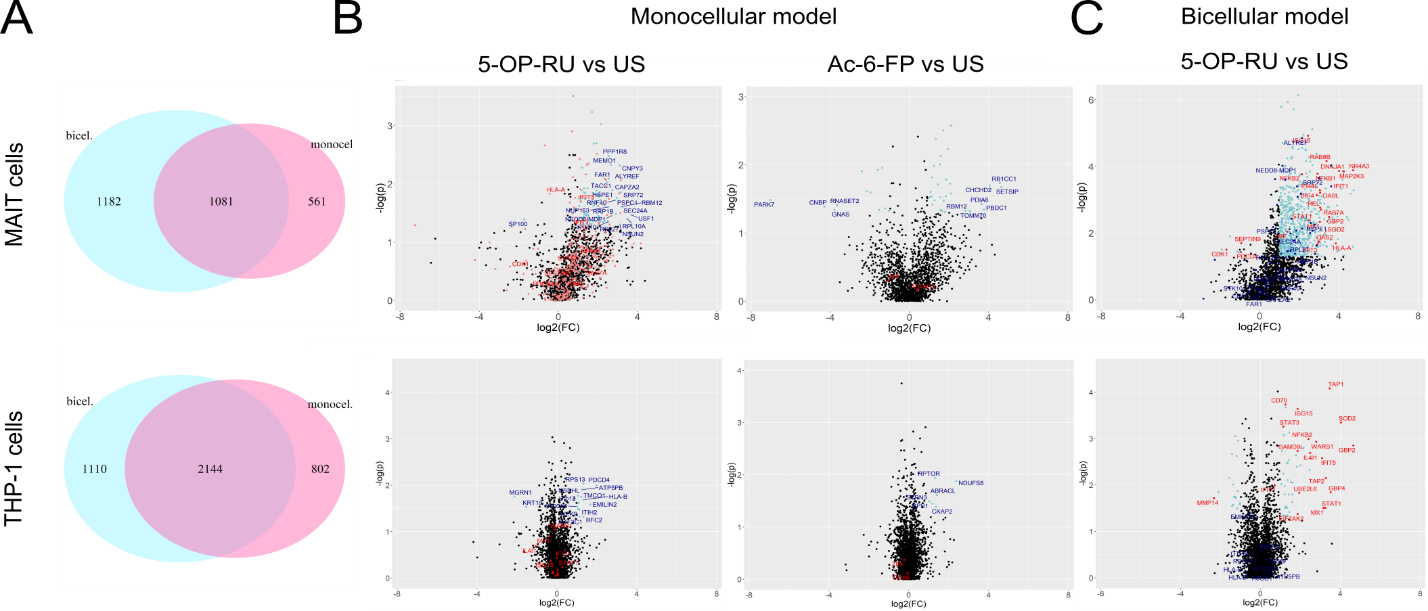
**

**Supplementary Figure S8: Comparison of differentially abundant proteins in mono- and bicellular system. A**) Venn diagrams showing identified proteins in mono- and bicellular system for MAIT or THP-1 cells. **B**) Volcano plots visualize differentially abundant proteins of primary human MAIT or THP-1 cells in the monocellular model. Differentially abundant proteins (p<0.05; log_2_(FC)>[1]) are highlighted in light blue. Proteins highlighted in red are the differentially abundant proteins from the bicellular model. Most abundant proteins from either mono- (dark blue) or bicellular model (red) are labeled with their respective gene names. **C**) Volcano plots showing protein abundances of MAIT and THP-1 cells from the bicellular model after 5-OP-RU stimulation. Proteins highlighted in light blue or labeled in red are differentially abundant proteins from the bicellular model while proteins highlighted in dark blue are differentially regulated in the monocellular model.

|  | **Stimulation** | **Upregulated** | **Downregulated** | **Proteins total** |
| --- | --- | --- | --- | --- |
| **MAIT cells** | 5-OP-RU | 108 | 1 | 1642 |
|  | Ac-6-FP | 31 | 12 |  |
| **THP-1 cells** | 5-OP-RU | 13 | 2 | 2946 |
|  | Ac-6-FP | 6 | 0 |  |

**Supplementary Table S9: Number of differentially abundant protein groups in the monocellular system after metabolite stimulation.** Total number of protein groups is given of proteins that were identified in at least one condition from three of four MAIT cell donors or two of three THP-1 replicates. Differentially abundant proteins were defined with p<0.05 and log_2_(FC)>[1].

**
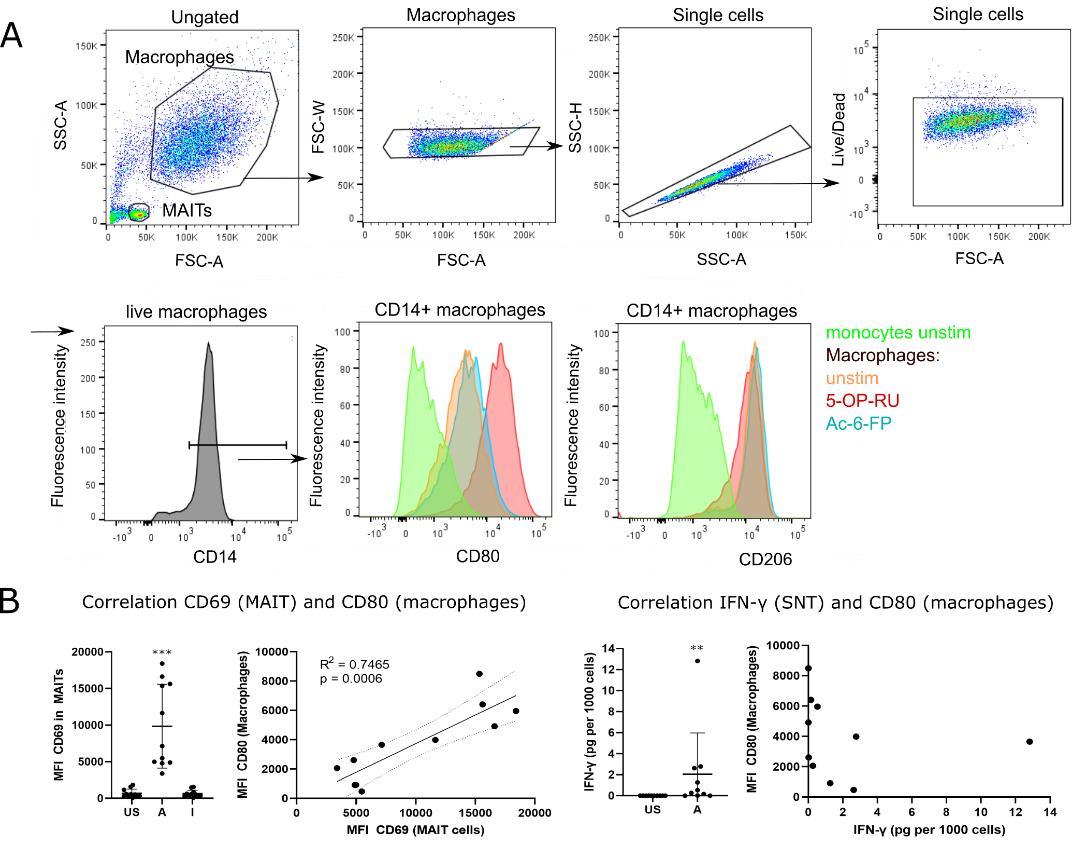
**

**Supplementary Figure S10:** **MAIT cell-induced M1 polarization of macrophages.** Primary M0 macrophages were stimulated with FACS-sorted MAIT cells of the same donor for 20 hours with 50 ng/ml 5-OP-RU or Ac-6-FP. **A**) Gating strategy for detection of CD80 and CD206 expression on primary macrophages is shown. Macrophages were discriminated from MAIT cells by forward scatter-area (FSC-A) and side scatter-area (SSC-A). Single macrophages were discriminated by forward scatter-area (FSC-A) and forward scatter-width (FSC-W) as well as side scatter-area (SSC-A) and side scatter-height (SSC-H). Live macrophages were discriminated with Live/Dead dye. CD80 and CD206 expression was determined by analyzing Median Fluorescence Intensity (MFI) in CD14^+^ macrophages. **B**) Correlation between MAIT cell activation and CD80 expression. CD69 was measured by flow cytometry on live, single cell MAIT cells. IFN-γ was determined by ELISA in supernatants (SNT) following stimulation. The median fluorescence intensity (MFI) of CD69 on MAIT or the amount of IFN-γ in the SNT was correlated with the MFI of CD80 on macrophages from the same donor. The solid line of the CD69/CD80 correlation shows a linear correlation with a 95% confidence interval (dotted lines). R square (R^2^) of linear regression and p-value for the scope to be non-zero are given. Asterisks indicate significant differences determined by Wilcoxon matched-pairs signed rank test; p** < 0.01: p***<0.001. Horizontal lines indicate mean ± SD. US = unstimulated; A = 50 ng/ml 5-OP-RU; I = 50 ng/ml Ac-6-FP; IFN = Interferon αB/D; FI = Fluorescence intensity.

**
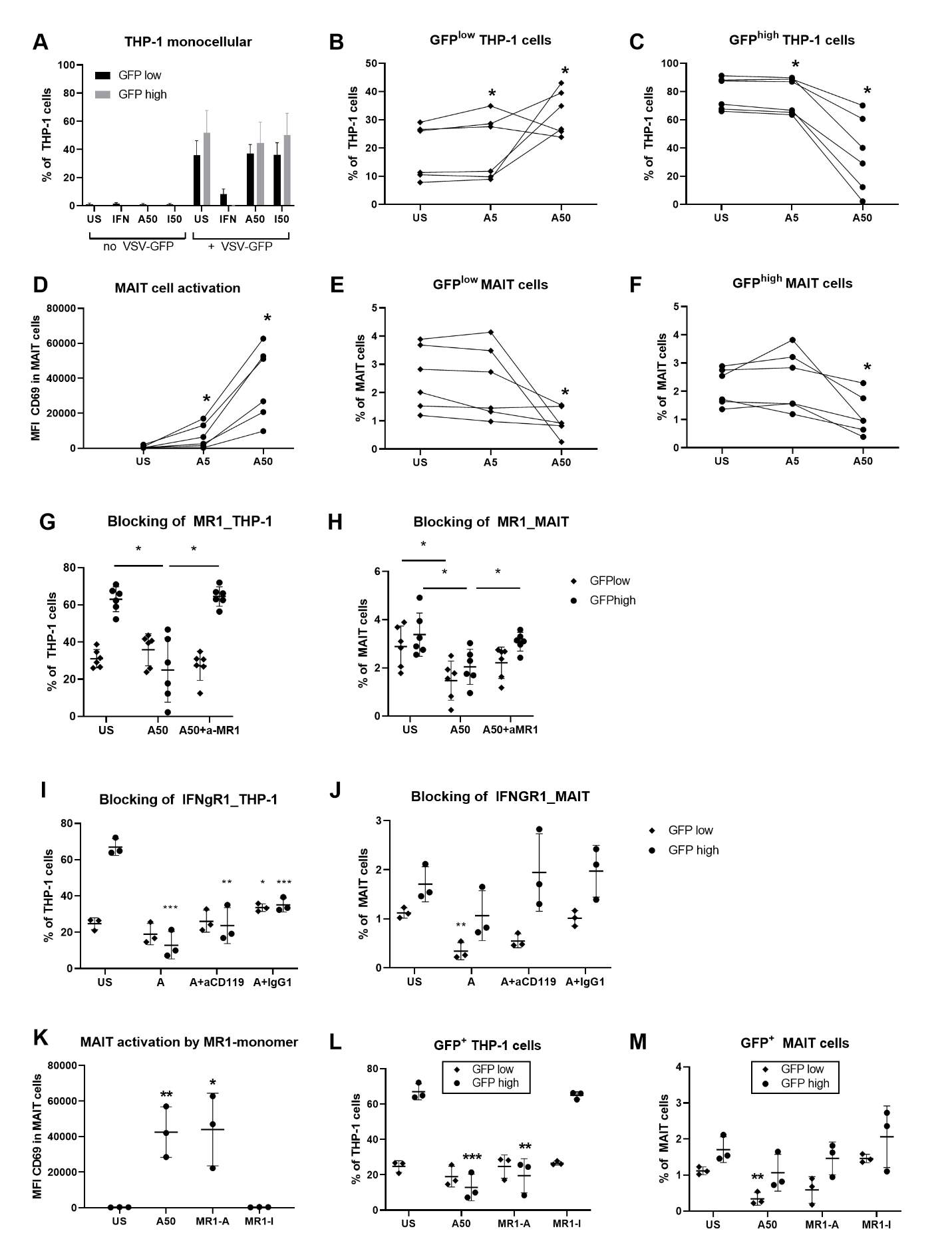
**

**Supplementary Figure S11: 5-OP-RU dose-dependent inhibition of virus replication in THP-1 and MAIT cells.** THP-1 cells alone (A) or THP-1 cells plus MAIT cells (B-F) were stimulated for 20 hours with 5-OP-RU, Ac-6-FP, or 500 U/ml Interferon-α/β (IFN). (D) MAIT cell activation (MFI CD69) was determined by flow cytometry after 20 hours. Pre-stimulated cells were infected with VSV-GFP (MOI 5) for 6 hours. Virus replication was quantified by measuring the GFP fluorescence by flow cytometry. (A) GFP signal before and after infection with VSV-GFP is shown. (B-H) GFP signal after 6 hours of infection with VSV-GFP is shown. (G+H) THP-1 cells were pre-incubated with 20 µg/ml anti-MR1 1 hour prior to stimulation with 5-OP-RU and MAIT cells. (I+J) MAIT and THP-1 cells were pre-incubated with 5 µg/ml anti-CD119 or Isoytpe control (IgG) 1 hour prior to stimulation with 5-OP-RU. (K-M) MAIT cells were pre-stimulated in MR1-monomer-coated wells before co-culturing them with naïve THP-1 cells for 20 hours and infecting them with VSV-eGFP. Data from three donors are shown. (A-H) Asterisks indicate significant differences determined by Wilcoxon matched-pairs signed rank test; p* < 0.05. Data from two independent experiments from six donors are shown. (I-M) Asterisks indicate significant differences determined by unpaired t-test. p* < 0.05; p** < 0.01; p***<0.001. US = unstimulated; A5 = 5 ng/ml 5-OP-RU; A50 = 50 ng/ml 5-OP-RU; I50 = 50 ng/ml Ac-6-FP; aMR1=anti-MR1; aCD119=anti-CD199; MR1-A/-I = 500 ng MR1-5-OP-RU/Ac-6-FP-monomer.
